# Evidence for High Levels of Gene Flow in Hedgehogs (*Erinaceus europaeus*) Across South Wales, UK, Despite Potential Anthropogenic and Natural Barriers to Dispersal

**DOI:** 10.1002/ece3.71201

**Published:** 2025-04-07

**Authors:** Samantha L. Shove, Lilith Zecherle Bitton, Simon Allen, Gabrielle M. K. Howell, Hazel J. Nichols

**Affiliations:** ^1^ Department of Biosciences Swansea University Swansea UK; ^2^ Cura Terrae Land and Nature W2 Business Centre Cardiff UK; ^3^ Geoteva Environmental Consultancy Omer Israel; ^4^ Gower Bird Hospital Swansea UK

**Keywords:** conservation management, dispersal barriers, gene flow, genetic structure, habitat resistance, hedgehogs, landscape connectivity, landscape resistance

## Abstract

Understanding how landscape connectivity affects gene flow can help to guide the management of animal species of conservation concern. One such species is the West European hedgehog ( *Erinaceus europaeus* ), which has seen significant declines across its distribution, with the highest rate of declines being reported in rural areas of the UK. The drivers of these declines are not well understood, but anthropogenic changes in the landscape such as modified agricultural practices and increased road traffic have been proposed to play a part. These impacts are likely to fragment populations into smaller sub‐populations, leading to genetic differentiation and depletion. Here, we used genetic (microsatellite) and landscape data to investigate the impact of habitat resistance and landscape features (roads and waterways) on the genetic structure of hedgehogs across a 5800 km^2^ area of South Wales, UK. We found evidence of weak genetic structuring, with four genetic clusters present across the study area, but many individuals were admixed. We found no evidence that this genetic structure was related to roads, waterways, habitat resistance, or geographic distance, suggesting that hedgehogs may be able to disperse across these potential barriers frequently enough to minimise genetic fragmentation. This study demonstrates the importance of understanding the interactions between a species and the wider landscape to inform conservation management.

## Graphical abstract

The West European Hedgehog ( *Erinaceus europaeus* ) has seen significant declines across its distribution, the drivers for which are not well understood, but anthropogenic changes in the landscape such as modified agricultural practices and increased road traffic have been proposed to play a part. These impacts are likely to fragment populations into smaller sub‐populations, leading to genetic differentiation and depletion. However, we found no evidence that this genetic structure was related to roads, waterways, habitat resistance, or geographic distance, suggesting that hedgehogs may be able to disperse across these potential barriers.

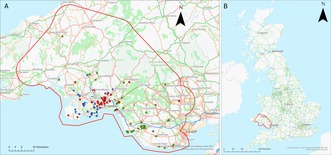

## 1 Introduction

Biodiversity has been in global decline over the last few thousand years, accelerating since the 1940s, with significant species and ecosystem declines seen across industrialised nations including the UK (Robinson and Sutherland 2002). These declines appear to be primarily driven by a combination of habitat loss (Andrén 1994; Stoate et al. 2001; Brooks et al. 2002; Crooks et al. 2017), habitat fragmentation (Bright 1993; Yanes et al. 1995; Fitzgibbon 1997; Clark et al. 2001; Van Dyck and Baguette 2005) and pollution (Stoate et al. 2001), but less well known factors may also contribute to declines, such as predation by pets (Baker et al. 2003; Dickman 1987; Robinson and Sutherland 2002) and transport fatalities (Coffin 2007).

In addition to causing population declines, these threats to biodiversity can also impact patterns of genetic diversity. For example, habitat fragmentation often leads to the separation of a single population into multiple smaller sub‐populations (Frankham et al. 2002; Allendorf et al. 2013). The increased rates of genetic drift in these sub‐populations can lead to them becoming genetically differentiated and depauperate (Frankham et al. 2002), which may in turn lead to an increased likelihood of inbreeding and reduced resilience to environmental change (Frankham et al. 2002; Allendorf et al. 2013). Understanding the distribution of genetic diversity therefore can help to reveal barriers to movement and gene flow, knowledge of which can aid in conservation decision making (Allendorf et al. 2013).

Western European hedgehogs ( *Erinaceus europaeus* ), referred to as hedgehogs from here on, have suffered major declines in recent decades, losing at least 60% of the population since the 1950s (Hof and Bright 2016; Morris 2018; Pettett et al. 2018; Finch et al. 2020). These declines are occurring across their range, but the highest rate of declines has been reported in rural areas of the UK (Araguas et al. 2022; Wembridge et al. 2022; Williams et al. 2018), making these populations a priority for further investigation. The drivers behind these declines are unclear and often debated but are likely to include habitat loss and fragmentation (Morris 2018; Moore et al. 2020; Taucher et al. 2020; Wright et al. 2020). In the UK, agricultural intensification and land use changes have resulted in increased field sizes, increased management frequency, the loss of field margins (Stoate et al. 2001; Robinson and Sutherland 2002), hedgerows/tree‐lines (Kotzageorgis and Mason 1997) and non‐agricultural habitat patches (Fitzgibbon 1997), all of which are known to provide connectivity and foraging habitats for hedgehogs (N. Reeve 1994; Hof and Bright 2010; van de Poel et al. 2015; Morris 2018). These habitat changes alter the suitability and resistance of habitats to hedgehog movement across the landscape, affecting the pattern of hedgehog movements as well as the availability and accessibility of the resources needed for survival (Driezen et al. 2007; Braaker et al. 2014; Wright et al. 2020).

Previous genetic studies on hedgehogs have found mixed results. Within urban areas, one study found that the presence of urban green spaces increased gene flow (Braaker et al. 2017), but others found relatively little genetic structure within single cities, possibly due to high connectivity (Barthel et al. 2020) or founder effects (Osaka et al. 2022). Those in rural areas found mixed structuring, often dependent on the scale of the study area and the presence of clear physical barriers such as mountains (Araguas et al. 2022) or water‐separated islands (Rasmussen et al. 2020). Others found weak structuring where a mix of movement barriers and corridors were present (Becher and Griffiths 1998; Curto et al. 2019), while Yu et al. (2024) showed little evidence of structuring across their four sub‐urban study sites. Henderson (2001) found some evidence of structuring due to distance within urban and rural areas around a UK city, but no relationship between genetic structure and habitat fragmentation. The degree to which the distribution of different habitats and features causes population fragmentation, and thus impacts gene flow and genetic diversity, is therefore currently unclear. This is particularly the case for more rural settings, where populations are still declining more rapidly (IUCN/CPSG 2023).

Hedgehog mortality and population fragmentation may also be influenced by infrastructure such as roads. Approximately 100,000 to 300,000 hedgehogs are killed on UK roads each year (Wright et al. 2020) so they contribute directly to population declines and may also act as a barrier to movement (Micol et al. 1994; Huijser and Bergers 2000; Rondinini and Doncaster 2002; Orłowski and Nowak 2004) with larger roads having a greater barrier effect (Orłowski and Nowak 2004; Hof and Bright 2009). The presence of watercourses may also present a barrier to hedgehog movements and may increase the effect of habitat fragmentation (Morris 2018). Accordingly, there is some evidence from genetic studies that large watercourses (particularly large rivers and straits between islands) act as a barrier to gene flow (Braaker et al. 2017; Rasmussen et al. 2020; Araguas et al. 2022). However, other studies have shown that hedgehogs can cross many waterbodies by using bridges and other structures or by swimming (Hof and Bright 2009; Barthel et al. 2020). As such, the barrier effect of watercourses on gene flow is unclear.

To minimise further declines and encourage recovery of hedgehog populations in the UK, it is vital to determine to what extent habitat resistance and the presence of roads and waterways have contributed to the potential physical and genetic isolation of hedgehog populations. Here, we used habitat resistance maps and microsatellite genetic data to investigate potential barriers to gene flow within and between a population of hedgehogs across South Wales, UK. We predicted that the population would have spatially distinct sub‐populations corresponding to the presence of high‐resistance habitats and natural and man‐made barriers to gene flow such as major watercourses and roads.

## 2 Methods

### 2.1 Study Species

Hedgehogs are generalist nocturnal mammals that feed on a wide range of invertebrates, small vertebrates and carrion, as well as taking advantage of human‐provided food in urban and sub‐urban environments (Dickman 1987; N. Reeve 1994; Braaker et al. 2014; Morris 2018). They are non‐territorial and have home ranges between 10 and 40 ha (Braaker et al. 2014), allowing them to take advantage of a variety of habitats and food sources (Driezen et al. 2007). Hedgehogs use a range of habitats from woodland and scrub to grassland and occasionally heath and have adapted to man‐made habitats such as parklands, field margins and hedgerows (N. Reeve 1994; Hof and Bright 2010; van de Poel et al. 2015; Morris 2018). They are now often more common in urban and sub‐urban areas than rural areas (Hof and Bright 2009; Hubert et al. 2011; Braaker et al. 2014; Williams et al. 2015; Pettett et al. 2017; Wilson and Wembridge 2018). Based on their known ecology, genetic differences within hedgehog populations are unlikely to be strongly influenced by territoriality, dispersal events, or very specialist species requirements, as has been shown in some other species (Baguette et al. 2013; Mateo‐Sánchez et al. 2015; Keeley et al. 2017).

### 2.2 Study Area

**FIGURE 1:**
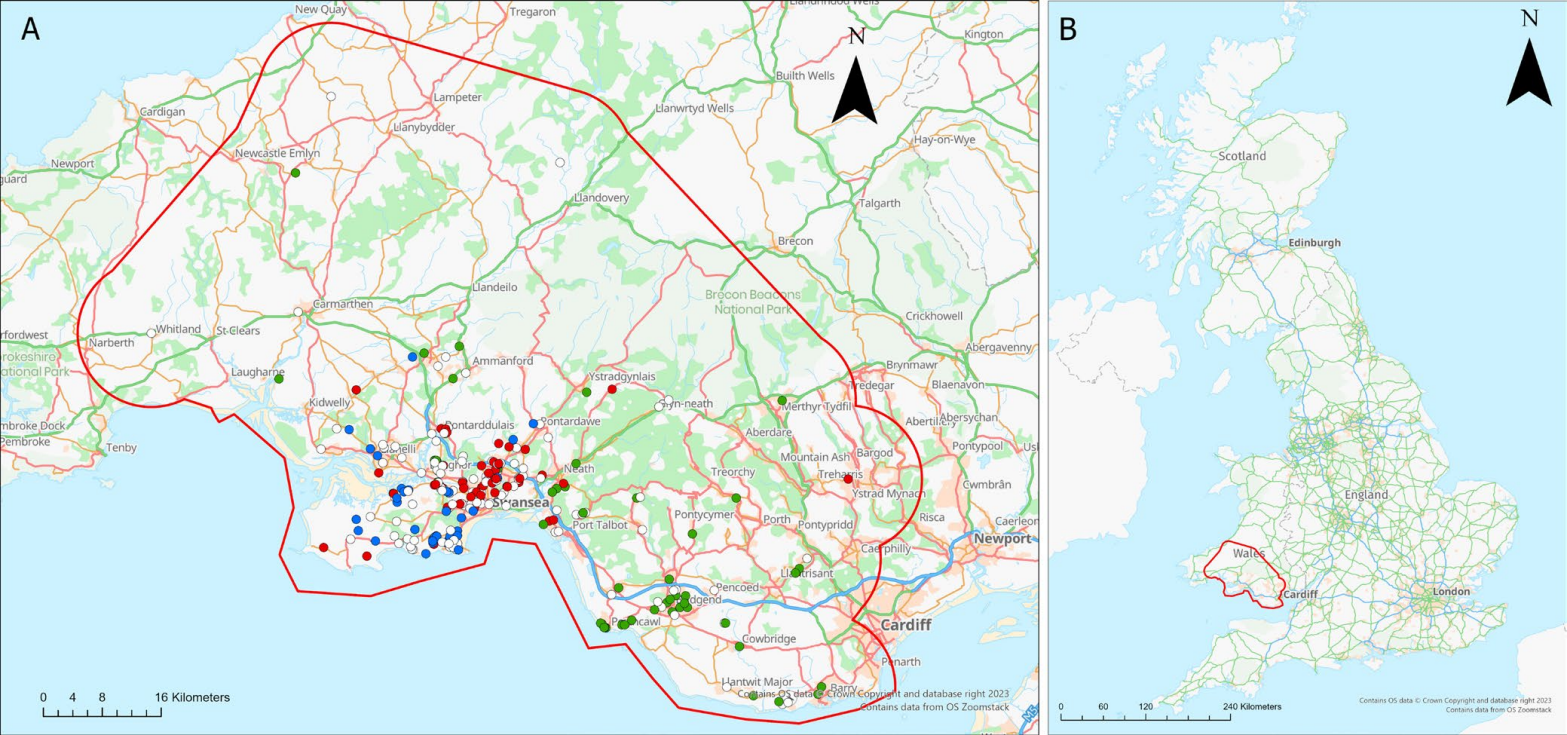
Mapping of the sampled individuals, along with their genetic cluster (*K* = 3): (A) For the full study area in South Wales (shown by the red line) (*K* = 3), (B) Shows the study area in the wider UK context. Major roads are shown in blue (M4) and green (A roads) and main rivers in light blue. The colours shown for individuals with > 0.7 assignment to a single cluster match those assigned in the structure analysis in Figure S2. Admixed individuals with < 0.7 assignment to a single cluster are shown in white.

The study area covered approximately 5800 km^2^ of south Wales (Figure 1). Hof and Bright (2012) identified that hedgehog sightings on farmland in Wales were among the lowest in the UK, while Williams et al. (2018) showed marked declines in hedgehog numbers in Wales in recent decades, making this area a priority for study. The study area was dominated by improved grassland habitat with notable areas of broadleaved plantation, dry heath/acid grassland mosaic and a mosaic (largely a combination of acid grassland, marshy grassland and wet and dry heath). The southern part of the study area included the urban and sub‐urban areas of Cardiff and Swansea and several major roadways including the M4 running east–west. Numerous watercourses were also present, including several large rivers running approximately north–south through the study area, namely, River Taff and River Ely to the east, Ogmore River, River Avan, River Neath and River Tawe to the centre and River Tywi, River Gwili and River Taf to the west.

The study area was defined by mapping the locations of origin of DNA‐sampled hedgehogs admitted to Gower Bird Hospital (GBH). The map was generated using ArcGIS Pro 3.0.3 (Inc 2023) using the grid references of each sample with a buffer of 10 km applied to produce a single merged buffer for all records. This buffer was then amended to remove isolated excluded pockets, to join across slivers of land and to follow coastlines. A 10 km buffer was chosen because displacement between hedgehog populations rarely exceeds this distance (Doncaster et al. 2001) and most hedgehog movements are under 5 km (N. J. Reeve 1981; N. Reeve 1994; Doncaster et al. 2001; Moorhouse et al. 2014; Morris 2018).

### 2.3 Genetic Analysis

DNA samples were taken by GBH staff from hedgehogs brought into GBH between October 2019 and September 2021. These samples included 147 buccal swabs and 164 tail tip or ear clip samples stored in ethanol and frozen at −20°C. Of these samples, 8 individuals had both buccal swabs and tail samples taken. Buccal swabs were taken while hedgehogs were under anaesthetic for veterinary procedures, while tail and ear samples were taken from hedgehogs that were dead on arrival or died while in the care of GBH. No hedgehogs were killed, handled, or anaesthetised for the purposes of this study. This study was conducted in accordance with the ethics permit from Swansea University, number 200721/213.

DNA extraction used the Qiagen DNeasy Blood and Tissue kit, following the manufacturer's instructions, applying the technique specified for tissue DNA extraction for both tissue and swab samples. This resulted in extracted DNA concentrations between 2.1 and 48.6 ng/μL with a mean of 15.19 ng/μL. Samples were genotyped using 14 fluorescently labelled microsatellite primers across three multiplex reactions (Table S1). The microsatellites were identified through published literature (Becher and Griffiths 1997; Henderson et al. 2000; Curto et al. 2019).

Polymerase chain reactions (PCR) were performed to amplify microsatellite sequences using a Qiagen Multiplex PCR Kit following the manufacturer's recommendations, except that we used 12 μL reaction volumes, to keep the use of reagents to a minimum. Each reaction contained 2 μL undiluted DNA and 0.2 μM primer. The following PCR conditions were used: one cycle of 15 min at 95°C; 35 cycles of 30 s at 94°C, 90 s at 57°C, 60 s at 72°C; and one final cycle of 30 min at 60°C. PCR products were resolved by electrophoresis on an ABI 3730xl capillary sequencer (Applied Biosystems) and allele sizes were scored using GeneMapper Software Version 4.0 (Applied Biosystems). To maximise genotype quality, we manually inspected all of the traces and corrected any genotype calls where necessary. To assess error rates for each microsatellite locus, we independently re‐genotyped a subset of 49 individuals and compared the resulting genotypes to calculate the error rate per allele. Summary statistics on the resultant genotypes and deviation from Hardy–Weinberg proportions were calculated using Cervus 3.0.7 (Kalinowski et al. 2007). Relatedness coefficients between all pairs of individuals were estimated using the GenAlEx Excel add‐in (Peakall and Smouse 2006; Peakall and Smouse 2012), following Queller and Goodnight (1989).

### 2.4 Population Structure Analysis

Structure 2.3.1 (Pritchard et al. 2010) was used to assess the genetic subdivision within the sample population. Structure uses a Bayesian approach to determine the most likely number of genetic clusters (*K*) within a sample population and the degree to which individuals belong to each cluster. *K* values ranging from 1 to 10 were subject to five independent runs, each with run length and MCMC of 10^5^ following a burn‐in time of 10^5^ using the admixture model. Given the difficulty in determining *K* values below 3 (Janes et al. 2017), the following *K* determinates were taken into account to assess the most likely value of *K*: (1) Average estimated likelihood of *K* (Ln Pr(X|*K*)), which estimates the posterior probability of the data across all runs, (2) estimated likelihood of *K* (Ln Pr(X|*K*)) for individual runs and (3) ΔK, the statistic developed by Evanno et al. (2005) based on the second‐order rate of change of LN Pr(X|*K*), as generated by Structure Selector Web Server (Li and Liu 2018), accessed 16 November 2024.

### 2.5 Habitat Resistance Mapping

To assess the impact of land use on the genetic structure of hedgehog populations, a habitat map of the study area was produced and a resistance value was allocated to each habitat type. Open‐source phase 1 habitat data were obtained from the LLE Geo‐Portal (accessed 03/04/2021), imported into ArcGIS and edited to cover the study area. Phase 1 habitat classification is a method for mapping and classifying habitats, including urban areas, developed by the Joint Nature Conservancy Council based on the floral species present, soil conditions and management practices (JNCC 2010).

The accuracy of the habitat mapping was checked against the latest aerial photography available within ArcGIS (dated from 2020), with corrections made as necessary. Those areas without a habitat code were checked against the aerial images and adjacent categorised phase 1 habitats, and a visual assessment was used to assign a habitat type. These checks were made at a scale of 1:5000, with all areas less than 1 ha not checked as they were likely too small to influence habitat resistance given the extent of the study area.

Once this mapping was completed, a habitat resistance field was added to the attribute table within ArcGIS, with each habitat type being assigned a resistance value category between 0 (low resistance) and 99 (high resistance/barrier) following (Zecherle et al. 2020). These values were based on species knowledge, such as resource requirements, need for cover, foraging behaviour and the identified habitat use patterns from previous studies (detailed within the SI: Tables S2 and S3). A similar process was followed for roads and watercourses which were classed into 6 and 4 categories, respectively, based on the ‘type’ information provided by the open‐source GIS data and associated likelihood of barrier effect (Tables S4 and S5). Improved grassland habitats were particularly challenging to classify given contradictory research results indicating different use levels (Tables S2 and S3). We selected a resistance of 31 (low‐medium) for this habitat, but we also tested six other resistance values ranging from 1 to 99. Overall, ten resistance layers were created: seven habitat resistance layers, barrier roads layer, barrier watercourse layer and a geographic distance control layer. These layers were converted in ArcGIS into raster maps using the ‘feature to raster’ tool with cell sizes set to 50 m in order to import the data into Circuitscape.

Circuitscape in Julia v1.8.4 (Anantharaman et al. 2020) was used to calculate the pairwise resistance distances for the sampled population against the different resistance surfaces. This approach utilises electronic circuit theory to estimate the resistance to current flow between nodes (sampled individuals) and was run in pairwise mode with nodes connected to all eight neighbouring cells (Zecherle et al. 2020).

### 2.6 Landscape Genetic Analysis

Downstream analyses were carried out in R version 4.3.1 (Team 2021). A distance‐based redundancy analysis (dbRDA) was used to test for a potential relationship between habitat/feature resistance distance and genetic distance. This followed (Zecherle et al. 2020) using the ‘capscale’ function in the ‘vegan’ package v2.6–4 (Legendre et al. 2011). This approach allows us to consider genetic distance as a response variable against which different explanatory variables can be regressed (Legendre and Anderson 1999; Buttigieg and Ramette 2014). To utilise this approach, the pairwise genetic distance matrices and the habitat/feature resistance matrices were first transformed into one‐dimensional response and explanatory variables by performing a Principal Coordinate Analysis (PCA) using the pcoa function in the ‘ape’ R package v5.7–1 with a Lingoes correction to address negative eigenvalues and preserve variation within the matrices (Paradis and Schliep 2018; Zecherle et al. 2020). The number of significant principal coordinates (PCos) to be retained was determined using a broken stick model (MacArthur 1957).

Separate dbRDA models were constructed with the transformed pairwise relatedness matrix set as the response variable and one of the transformed resistance matrices (geographic distance, habitat, roads, and waterbodies) set as the explanatory variable. Models were also tested that controlled for an effect of geographic distance on habitat/feature resistance. Finally, we ran a model including all of the explanatory variables together. All models were tested for significance using the anova.cca function with 9999 permutations.

## 3 Results

### 3.1 Genetic Analysis

Of 303 individual samples obtained, 298 samples were successfully genotyped, 98.3% of the samples provided by GBH. Our panel of 14 microsatellites had a high level of diversity and information content; mean observed heterozygosity of the microsatellites within the sampled population was 0.656 (range 0.336–0.801), mean Polymorphic Information Content (PIC) was 0.614 (range 0.327–0.761) and the mean number of alleles per locus was 7.786 (range 3–11) (Table 1). There was no significant deviation from the Hardy–Weinberg equilibrium for any locus (Table 1). The estimated frequency of null alleles was under 0.1 for all loci and was under 0.05 for all but three, the highest having an estimated frequency of 0.067 (Table 1). As these estimated null allele rates were low and other loci registered successfully during PCR processing, the potential presence of null alleles is likely to have a negligible effect on population‐genetic parameters, so all loci were used in further analysis (Allendorf et al. 2013, 93–94).

**TABLE 1:** Genotyping summary. Displayed are the loci used, number of alleles associated with each, number of hedgehogs genotyped using that locus, observed and expected heterozygosity, polymorphic information content (PIC), deviation from Hardy–Weinberg expected proportions of genotypes (HW; NS represents not significant) and estimated frequency of null alleles.

Locus	No. of alleles	No. of hedgehog genotyped	Observed heterozygosity	Expected heterozygosity	PIC	HW	Estimated frequency of null alleles
E13	9	298	0.728	0.792	0.761	NS	0.041
EEU1	8	300	0.407	0.403	0.35	NS	−0.012
EEU12H	3	300	0.377	0.42	0.359	NS	0.052
EEU37H	6	295	0.336	0.343	0.327	NS	0.001
EEU4	7	298	0.728	0.783	0.748	NS	0.035
EEU2	9	302	0.636	0.712	0.666	NS	0.056
EEU3	7	298	0.614	0.639	0.581	NS	0.016
EEU5	11	299	0.712	0.767	0.731	NS	0.036
EEU6	6	302	0.586	0.669	0.609	NS	0.067
E36	7	301	0.801	0.781	0.748	NS	−0.016
EEU43H	10	298	0.695	0.72	0.685	NS	0.020
W23	8	285	0.586	0.629	0.573	NS	0.035
W30	10	302	0.689	0.75	0.72	NS	0.044
W8	8	301	0.721	0.77	0.732	NS	0.033

A subset of 49 samples was re‐genotyped and the two sets compared to determine the error level within the genotyped data. The error rate was zero for 12 of the loci and was very low (with a rate of < 0.04 inconsistencies per allele) for the remaining two loci (Table S6). Furthermore, three of the individuals where both tail and swab samples were taken were successfully genotyped and showed no variation in results, demonstrating that genotypes from non‐invasive swabs were consistent with those from tissue samples.

### 3.2 Population Structure Analysis

The highest average Ln Pr(X|*K*) generated by Structure during the five independent runs for each *K* value indicated a *K* value of 3, although there is only a slight difference between this and *K* = 4 (Figure S1). The Structure Selector analysis indicated a *K* value of 3 based on the mean LnP(*K*), just above that for *K* = 4 (Table S7) while ΔK showed a strong peak at *K* = 4. Overall, the analysis indicates that there are most likely 3 genetic clusters within the sample population.

The Structure ancestry analysis (Figures 1 and S7) based on *K* = 3 shows a clear genetic cluster assigned for 155 (52%) individuals, while 143 (48%) could not be assigned to a single cluster (*q*‐values < 0.7). Mapping the likely clusters based on *K* = 3 (Figure 1 and S7) shows the overlap between the clusters, with one largely limited to the Gower peninsula (green cluster), one largely in the Swansea area (red cluster) and another spread around the Swansea/Bridgend area but also with several individuals to the north and east (yellow cluster). The remaining individuals, shown in white, could not be clearly assigned to a specific cluster and are spread throughout the study area.

### 3.3 Landscape Genetic Analysis

The PCA and broken stick model indicated that the first 10 PCos should be retained for the distance control, roads and water resistance matrices; these accounted for > 50% of the percentage variance within the samples. The first 10 PCos for the habitat resistance matrix only accounted for > 4% of the percentage variance within the samples, and 131 PCos were required to account for > 50% of the percentage variance. As such, the first 10 PCos were used within the transformed resistance matrices for the distance control, roads and water variables, and 131 PCos were used for the habitat resistance variables for consistency (Table S8).

None of the explanatory variables had a statistically significant effect on pairwise genetic relatedness, either alone or when controlled for geographic distance (Table 2). Altering the habitat resistance value of improved grassland did not qualitatively affect our results, and neither did replacing the Queller and Goodnight (1989) relatedness estimator with the Lynch and Ritland (1999) estimator (Tables S9 and S10).

**TABLE 2:** Results from the dbRDA investigating the impact of resistance variables on pairwise genetic relatedness (Queller and Goodnight (1989) relatedness estimator). Displayed are the variables tested, their total variance (inertia), the % variance explained (*R* ^2^) and adjusted % variance explained (adjusted *R* ^2^), the degrees of freedom (df), *F*‐statistic (*F*) and *p*‐value (Pr(>F)) of the permutation tests (9999). Models controlled for geographical distance are indicated with Habitat resistance is based on improved grassland (IG) having a resistance value of 31.

Variable	Inertia	*R* ^2^	Adjusted *R* ^2^	df	*F*	Pr(>F)
Distance	8.82	3.75%	< 1%	10	0.995	0.688
Roads	8.83	3.76%	< 1%	10	0.996	0.653
Water	8.82	3.75%	< 1%	10	0.995	0.693
Habitats (IG Resistance = 31)	116.05	49.37%	< 1%	131	0.997	0.886
Roads|Distance	8.82	3.78%	< 1%	10	1.002	0.452
Water|Distance	8.82	3.77%	< 1%	10	1.000	0.525
Habitats (IG Resistance = 31)|Distance	8.82	49.36%	< 1%	131	0.997	0.800
All variables (IG resistance = 31)	235.06	60.71%	< 1%	161	0.998	0.723

## 4 Discussion

We found a weak genetic structure within the hedgehog population across our study area, with four genetic clusters; one being primarily found in the south east, another primarily on the Gower peninsula and the remaining two clusters around the Swansea area. However, over half of the individuals sampled were not clearly allocated to a specific cluster, demonstrating relatively weak genetic structure. Patterns of genetic relatedness across the study area were seemingly unrelated to geographic distance, habitat resistance, or the presence of large barrier features (rivers and roads) within the landscape.

### 4.1 The Impact of Habitat and Feature Resistance on Gene Flow

While previously published research based on individually tracking hedgehogs demonstrates that the species avoids certain habitats (Driezen et al. 2007), this does not appear to have affected patterns of relatedness across the study area. This was repeated across all habitat resistance models despite variations in the resistance levels used for improved grassland habitat, which dominated the study area. This suggests that hedgehogs may cross higher resistance habitats with sufficient frequency to allow gene flow across South Wales. Alternatively, habitat resistance may impact movement, but there may be other drivers of hedgehog movements (not measured in this study) that override the habitat resistance as a factor determining gene flow, such as availability of resting or nesting habitat, disturbance, food availability and risk of predation (Doncaster 1993; Doncaster et al. 2001; Riber 2006; Driezen et al. 2007; Berger et al. 2020), all factors identified by the Hedgehog Conservation Strategy (IUCN/CPSG 2023) within the healthy hedgehog habitat research priority. Similar patterns have been found in some other mobile mammals. For example, in Dall's sheep *Ovis dalli dalli* (Roffler et al. 2016) and Asiatic wild ass *Equus hemionus* (Zecherle et al. 2020), habitat resistance impacts habitat use but not gene flow, likely due to relatively high mobility through areas of non‐preferred habitats. However, mobility does not always negate the impacts of habitat resistance, with genetic structuring in relation to habitat sometimes being found in species with substantial dispersal capabilities, such as the grey long‐eared bat *Plecotus austriacus* (Razgour et al. 2014) and pine marten *Martes martes* (Ruiz‐González et al. 2014). It is also possible that our methods of classifying habitat resistance (based on previous literature) were insufficiently informative to uncover impacts on genetic structure, and some differences in habitat suitability (e.g., differing agricultural methods and intensity) may be difficult to identify using aerial imagery. Future studies using GPS data to assess hedgehog habitat use across our study area may be able to reveal subtle impacts of habitat fragmentation on fine‐scale genetic structure (Braaker et al. 2017; Müller et al. 2023).

Contrary to expectations, the presence of roads was found to have no significant impact on genetic relatedness within our study area. Roads present substantial features within the landscape, including motorways and busy ‘A' roads, and could be expected to have some form of barrier effect (Orłowski and Nowak 2004; Moore et al. 2020). Indeed, road barriers to gene flow have been found in some other mammals such as the European wildcat *Felis silvestris* (Westekemper et al. 2021), wild boar *Sus scrofa meridionalis* (Lecis et al. 2022) and red deer *Cervus elaphus* (Frantz et al. 2012). However, our results suggest that hedgehogs are able to cross or circumvent the majority of roads in our study area, including the M4 motorway, in sufficient numbers to maintain genetic connectivity, which does not need to be large. This is consistent with some previous studies suggesting that hedgehogs may have to some degree adapted their behaviour to the presence of roads, with hedgehogs increasing their movement speed when crossing roads (Doncaster et al. 2001) and adapting their behaviour to traffic patterns, becoming active later in the day when traffic levels are reduced (Dowding et al. 2010). Even large roads may present a limited barrier to movement: Doncaster (1992) observed individual hedgehogs in London crossing major trunk roads, and reports of hedgehogs successfully crossing or circumventing roads also occur in other studies (Doncaster et al. 2001; Dowding et al. 2010; Braaker et al. 2014; Williams et al. 2018; Barthel et al. 2020). Roads have even been reported to aid hedgehog movements in some studies, depending on the suitability of the verge habitats associated with them (Doncaster et al. 2001; Rondinini and Doncaster 2002; Hof and Bright 2009; Hof and Bright 2012; Wright et al. 2020) and the presence of crossing structures such as underpasses and culverts (Moore et al. 2020). This may also explain why some population genetic studies, ours included, have not identified roads as barriers to gene flow, even in urban areas with high traffic densities (Barthel et al. 2020). It should also be noted that our panel of 14 microsatellite markers (or genetic markers more generally) may not be sufficient to detect barrier effects from roads on a consistent basis. Nevertheless, the presence of gene flow does not negate the risk of demographic isolation, and roads are likely to cause substantial mortality in hedgehogs when they are crossed, so they are likely to impact on population declines (Wright et al. 2020).

The presence of several large watercourses was also found to have no significant impact on genetic relatedness within our study area. This is not wholly unexpected given that several studies indicate that hedgehogs are able to cross such features (Doncaster 1992). However, given the high number of watercourses present, including several large rivers, some effect on genetic relatedness could be expected, as has been found in some other studies of hedgehog population genetics (Braaker et al. 2017; Rasmussen et al. 2020; Araguas et al. 2022). As with roads, the lack of significant impact suggests that hedgehogs are able to cross rivers in South Wales, either by swimming, circumventing them or using finer scale connectivity features to cross that are not accounted for within the models used, including man‐made bridges (Barthel et al. 2020).

### 4.2 Genetic Structure

The weak genetic structure identified within the sample population was unexpected given the size of the study area and published research indicating that habitat resistance, roads and watercourses influence hedgehog movement (Driezen et al. 2007; Braaker et al. 2014; Morris 2018; Wright et al. 2020). However, weak population structure is consistent with some other studies operating on a similar scale (e.g., Bolfíková et al. 2013; Rasmussen et al. 2019; Barthel et al. 2020). The influence of geographic distance on the genetic structuring has also been shown to be slight, if present at all, by Becher and Griffiths (1998), Braaker et al. (2017), Curto et al. (2019) and Barthel et al. (2020), which indicates that the lack of a significant distance effect within our sample population is relatively common with hedgehogs over a variety of spatial scales. These studies suggest that other factors are likely to affect gene flow, such as geographic barriers, differences in temporal and spatial scaling of restricting factors and the influence of released animals on genetic mixing.

In contrast, some other studies have shown slight to moderate but significant genetic structure at smaller and larger scales than our study (Braaker et al. 2017; Curto et al. 2019; Rasmussen et al. 2020; Araguas et al. 2022). This suggests that our sample population has a higher rate of gene flow than some other populations. It is noted that the study by Rasmussen et al. (2020) covered several islands which are physically more isolated than our study area. Both the Araguas et al. (2022) and Curto et al. (2019) studies included significant mountain ranges between populations, which are not reflected within our study area. It is also noted that the Braaker et al. (2017) study covered a smaller and largely urban area while our study area was larger and largely rural. It is also possible that the genetic structure of hedgehogs in South Wales is yet to reflect recent changes in anthropogenic habitat resistance and barrier effects in the landscape. However, it is unlikely that a time lag is responsible for the lack of significant influence of rivers and roads as many of these have been present for at least several decades, including the M4 motorway which was completed in the 1980s (Coulon et al. 2006; Balkenhol et al. 2016; Lecis et al. 2022).

A further driver of high levels of gene flow in our study could be changing land use patterns. For example, when comparing the 2016 habitat mapping and the more recent 2020 aerial imagery used in our study, over 31.65km^2^ (approximately 0.5% of the overall study area) had been converted from semi‐natural habitats to urban/sub‐urban use. Such disturbance may result in the migration of animals away from these areas into adjacent habitats (Abu Baker et al. 2017; Tarabon et al. 2019) particularly during the clearance and construction phases. High levels of widespread migration induced by short term/rapid land‐use change might also influence the level of admixture observed, whereby over 50% of the sample population could not be assigned to a single genetic cluster.

Finally, the release of animals from rescue and rehabilitation centres may also be contributing to the level of admixture within the sample population (Barthel et al. 2020; Ploi et al. 2020; Araguas et al. 2022) particularly where animals are not released to the same location that they were rescued from. Jensen et al. (2017) studied harbour seal ( *Phoca vitulina* ) rehabilitation and showed this to have negative implications for the genetic health and success of a population by introducing inbred individuals or those less genetically suited to an area, although it was not considered to be a significant risk to wild populations. However, Pacioni et al. (2017) demonstrated that the release of rehabilitated woylie (*Bettongia penicillate*) individuals can benefit wild populations by counteracting genetic drift and boosting genetic diversity. The occurrence of uncoordinated translocations for many species is not quantified or recorded at present (Pyke and Szabo 2018; Barthel et al. 2020), but hedgehogs are one of the most commonly admitted species to rescues in the UK (Molony et al. 2006). This could result in their populations being particularly impacted by translocations. Although the GBH does release hedgehogs back to the locations they were found in, not all rescues do, location data may not always be available when animals are admitted by the public (Molony et al. 2006).

Genetic isolation has recently been identified as one of six key threats by Britain's National Hedgehog Conservation Strategy IUCN/CPSG (2023) as well as a priority for further research. Our results suggest that the threat of genetic isolation due to habitat type, roads and rivers may not be as significant an issue for hedgehogs as other factors, at least over our study area in south Wales. However, geographic areas with other landscape features such as islands and mountain ranges may show greater genetic fragmentation (Rasmussen et al. 2020; Araguas et al. 2022). Furthermore, smaller features that facilitate or reduce dispersal (such as hedgerows, verges and connectedness of residential gardens) that were present at too fine a scale for inclusion in this study may be better predictors of gene flow and should be investigated further in future. Being able to identify which features result in genetic isolation and which features encourage hedgehog movement is a key part in ensuring effective conservation management efforts in the future (IUCN/CPSG 2023).

## 5 Conclusion

Our study investigated potential barriers to gene flow in wild hedgehogs through combining genetic data from almost 300 individuals with knowledge of landscape features and habitat suitability across a 5800 km^2^ area of south Wales, UK. We found evidence of relatively weak genetic sub‐structuring of the population, but this did not correspond to patterns of isolation by distance, nor the presence of high‐resistance habitats or natural and man‐made barriers to gene flow such as major watercourses and roads. This suggests that, despite resulting in recent population declines, anthropogenic influences are not currently generating insurmountable barriers to gene flow. This may be due to hedgehogs being capable of regular dispersal across unfavourable habitat. But this could also be influenced by anthropogenic disturbance inducing dispersal, or rehabilitation practices increasing population admixture. What is not clear is whether the structure we observed represents a stable genetic equilibrium within the study area, or whether changes are still in process that may result in more defined population clustering or increased admixture in future.

## Author Contributions

**Samantha L. Shove:** conceptualization (equal), data curation (lead), formal analysis (lead), funding acquisition (supporting), investigation (equal), methodology (equal), project administration (equal), resources (equal), software (lead), visualization (lead), writing – original draft (lead), writing – review and editing (equal). **Lilith Zecherle Bitton:** methodology (equal), software (supporting), writing – review and editing (equal). **Simon Allen:** conceptualization (equal), resources (equal), writing – review and editing (equal). **Gabrielle M. K. Howell:** investigation (equal), writing – review and editing (equal). **Hazel J. Nichols:** conceptualization (equal), data curation (supporting), funding acquisition (lead), methodology (equal), project administration (equal), resources (equal), supervision (lead), validation (lead), writing – review and editing (equal).

## Conflicts of Interest

The authors declare no conflicts of interest.

## Supporting information


Data S1.

Data S2.

## Data Availability

All the required data are uploaded as Supporting Information.
