# Supplementary material for: Evidence for High Levels of Gene Flow in Hedgehogs (Erinaceus europaeus) Across South Wales, UK, Despite Potential Anthropogenic and Natural Barriers to Dispersal
Source: Ecol Evol. 2025 Apr 7;15(4):e71201. doi: 10.1002/ece3.71201 (PMC11975622; doi:10.1002/ece3.71201)
Supplement: Supplementary file 1 — Data S1. [file ECE3-15-e71201-s001.zip › ece371201-sup-0040-R Code.docx]

# load vegan and ape packages

# load ggplot2

# create explanatory variables from resistance matrices (Circuitscape data) using weighted PCoA

# import explanatory data as matrices

# note: hab labels contain number ones not lowercase letter L

distance<-read.table("distance.txt", header=TRUE, row.names=1)

habg1<-read.table("habg1.txt", header=TRUE, row.names=1)

habg11<-read.table("habg11.txt", header=TRUE, row.names=1)

habg31<-read.table("habg31rf.txt", header=TRUE, row.names=1)

habg51<-read.table("habg51.txt", header=TRUE, row.names=1)

habg71<-read.table("habg71.txt", header=TRUE, row.names=1)

habg91<-read.table("habg91.txt", header=TRUE, row.names=1)

habg99<-read.table("habg99.txt", header=TRUE, row.names=1)

road<-read.table("roads.txt", header=TRUE, row.names=1)

water<-read.table("water.txt", header=TRUE, row.names=1)

############################################################################

# import response data as matrices (pairwise genetic info generated through GenAlEx)

# LRM - Lynch & Ritland (1999) estimator - Mean

# QGM - Queller and Goodnight (1989) estimator - Mean

pwiselrm<-read.table("pairwiselrm.txt", header=TRUE, row.names=1)

pwiseqgm<-read.table("pairwiseqgm.txt", header=TRUE, row.names=1)

# transform into distance matrices

distancea<-as.dist(distance)

habg1a<-as.dist(habg1)

habg11a<-as.dist(habg11)

habg31a<-as.dist(habg31)

habg51a<-as.dist(habg51)

habg71a<-as.dist(habg71)

habg91a<-as.dist(habg91)

habg99a<-as.dist(habg99)

roada<-as.dist(road)

watera<-as.dist(water)

pwiselrma<-as.dist(pwiselrm)

pwiseqgma<-as.dist(pwiseqgm)

############################################################################

# PCoAs

# perform PCoA for each explanatory variable (distance, habitats, roads, and water)

pcoadist<-pcoa(distancea, correction="lingoes", rn=NULL)

pcoahabg1<-pcoa(habg1a, correction="lingoes", rn=NULL)

pcoahabg11<-pcoa(habg11a, correction="lingoes", rn=NULL)

pcoahabg31<-pcoa(habg31a, correction="lingoes", rn=NULL)

pcoahabg51<-pcoa(habg51a, correction="lingoes", rn=NULL)

pcoahabg71<-pcoa(habg71a, correction="lingoes", rn=NULL)

pcoahabg91<-pcoa(habg91a, correction="lingoes", rn=NULL)

pcoahabg99<-pcoa(habg99a, correction="lingoes", rn=NULL)

pcoaroad<-pcoa(roada, correction="lingoes", rn=NULL)

pcoawater<-pcoa(watera, correction="lingoes", rn=NULL)

pcoadist$values

pcoahabg1$values

pcoahabg11$values

pcoahabg31$values

pcoahabg51$values

pcoahabg71$values

pcoahabg91$values

pcoahabg99$values

pcoaroad$values

pcoawater$values

############################################################################

# identify number of PCos to retain - those above red line

# habitats code amended to reflect pcoa columns, bar [,3] and line [,4]

# distance

df.bar<-barplot(pcoadist$values[,2], xlab="PCos", ylab="percentage variation explained")

lines(x=df.bar, y=pcoadist$values[,3], col='red')

# habitatsGL1

df.bar<-barplot(pcoahabg1$values[,3], xlab="PCos", ylab="percentage variation explained")

lines(x=df.bar, y=pcoahabg1$values[,4], col='red')

# habitatsGL11

df.bar<-barplot(pcoahabg11$values[,3], xlab="PCos", ylab="percentage variation explained")

lines(x=df.bar, y=pcoahabg11$values[,4], col='red')

# habitatsGL31

df.bar<-barplot(pcoahabg31$values[,3], xlab="PCos", ylab="percentage variation explained")

lines(x=df.bar, y=pcoahabg31$values[,4], col='red')

# habitatsGL51

df.bar<-barplot(pcoahabg51$values[,3], xlab="PCos", ylab="percentage variation explained")

lines(x=df.bar, y=pcoahabg51$values[,4], col='red')

# habitatsGL71

df.bar<-barplot(pcoahabg71$values[,3], xlab="PCos", ylab="percentage variation explained")

lines(x=df.bar, y=pcoahabg71$values[,4], col='red')

# habitatsGL91

df.bar<-barplot(pcoahabg91$values[,3], xlab="PCos", ylab="percentage variation explained")

lines(x=df.bar, y=pcoahabg91$values[,4], col='red')

# habitatsGL99

df.bar<-barplot(pcoahabg99$values[,3], xlab="PCos", ylab="percentage variation explained")

lines(x=df.bar, y=pcoahabg99$values[,4], col='red')

# roads

df.bar<-barplot(pcoaroad$values[,2], xlab="PCos", ylab="percentage variation explained")

lines(x=df.bar, y=pcoaroad$values[,3], col='red')

# water

df.bar<-barplot(pcoawater$values[,2], xlab="PCos", ylab="percentage variation explained")

lines(x=df.bar, y=pcoawater$values[,3], col='red')

############################################################################

# variation explained by first PCos, relative eigenvalues / relative correlated evs for habs

# habitats code amended to reflect pcoa columns, [,3]

sum(pcoadist$values[1:1,2])

sum(pcoahabg1$values[1:1,3])

sum(pcoahabg11$values[1:1,3])

sum(pcoahabg31$values[1:1,3])

sum(pcoahabg51$values[1:1,3])

sum(pcoahabg71$values[1:1,3])

sum(pcoahabg91$values[1:1,3])

sum(pcoahabg99$values[1:1,3])

sum(pcoaroad$values[1:1,2])

sum(pcoawater$values[1:1,2])

############################################################################

# variation explained by first 4 PCos, relative eigenvalues / relative correlated evs for habs

# habitats code amended to reflect pcoa columns, [,3]

sum(pcoadist$values[1:4,2])

sum(pcoahabg1$values[1:4,3])

sum(pcoahabg11$values[1:4,3])

sum(pcoahabg31$values[1:4,3])

sum(pcoahabg51$values[1:4,3])

sum(pcoahabg71$values[1:4,3])

sum(pcoahabg91$values[1:4,3])

sum(pcoahabg99$values[1:4,3])

sum(pcoaroad$values[1:4,2])

sum(pcoawater$values[1:4,2])

############################################################################

# variation explained by first 9 PCos, relative eigenvalues / relative correlated evs for habs

# habitats code amended to reflect pcoa columns, [,3]

sum(pcoadist$values[1:9,2])

sum(pcoahabg1$values[1:9,3])

sum(pcoahabg11$values[1:9,3])

sum(pcoahabg31$values[1:9,3])

sum(pcoahabg51$values[1:9,3])

sum(pcoahabg71$values[1:9,3])

sum(pcoahabg91$values[1:9,3])

sum(pcoahabg99$values[1:9,3])

sum(pcoaroad$values[1:9,2])

sum(pcoawater$values[1:9,2])

############################################################################

# variation explained by first 10 PCos, relative eigenvalues / relative correlated evs for habs

# habitats code amended to reflect pcoa columns, [,3]

sum(pcoadist$values[1:10,2])

sum(pcoahabg1$values[1:10,3])

sum(pcoahabg11$values[1:10,3])

sum(pcoahabg31$values[1:10,3])

sum(pcoahabg51$values[1:10,3])

sum(pcoahabg71$values[1:10,3])

sum(pcoahabg91$values[1:10,3])

sum(pcoahabg99$values[1:10,3])

sum(pcoaroad$values[1:10,2])

sum(pcoawater$values[1:10,2])

# create matrices from first 1/10 (corrected) PCos

dist<-(pcoadist$vectors[,1:10])

habsg1<-(pcoahabg1$vectors[,1:10])

habsg11<-(pcoahabg11$vectors[,1:10])

habsg31<-(pcoahabg31$vectors[,1:10])

habsg51<-(pcoahabg51$vectors[,1:10])

habsg71<-(pcoahabg71$vectors[,1:10])

habsg91<-(pcoahabg91$vectors[,1:10])

habsg99<-(pcoahabg99$vectors[,1:10])

roads<-(pcoaroad$vectors[,1:10])

waters<-(pcoawater$vectors[,1:10])

############################################################################

# run dbRDA models - one per explanatory variable

# set 1 - LRM pairwise models (ma)

# pairwise matrix as response variable - distance matrices created at start

# distance, habitats, roads, and water as explanatory variables - PCos matrices created above

# ma1 - pairwise and distance

ma1<-capscale(pwiselrma~dist, add="lingoes")

print(ma1)

anova.cca(ma1,permutations=how(nperm=9999))

# ma2 - pairwise and habitatGL1

ma2<-capscale(pwiselrma~habsg1, add="lingoes")

print(ma2)

anova.cca(ma2,permutations=how(nperm=9999))

# ma3 -pairwise and habitatGL11

ma3<-capscale(pwiselrma~habsg11, add="lingoes")

print(ma3)

anova.cca(ma3,permutations=how(nperm=9999))

# ma4 -pairwise and habitatGL31

ma4<-capscale(pwiselrma~habsg31, add="lingoes")

print(ma4)

anova.cca(ma4,permutations=how(nperm=9999))

# ma5 -pairwise and habitatGL51

ma5<-capscale(pwiselrma~habsg51, add="lingoes")

print(ma5)

anova.cca(ma5,permutations=how(nperm=9999))

# ma6 -pairwise and habitatGL71

ma6<-capscale(pwiselrma~habsg71, add="lingoes")

print(ma6)

anova.cca(ma6,permutations=how(nperm=9999))

# ma7 -pairwise and habitatGL91

ma7<-capscale(pwiselrma~habsg91, add="lingoes")

print(ma7)

anova.cca(ma7,permutations=how(nperm=9999))

# ma8 -pairwise and habitatGL99

ma8<-capscale(pwiselrma~habsg99, add="lingoes")

print(ma8)

anova.cca(ma8,permutations=how(nperm=9999))

# ma9 - pairwise and roads

ma9<-capscale(pwiselrma~roads, add="lingoes")

print(ma9)

anova.cca(ma9,permutations=how(nperm=9999))

# ma10 - pairwise and water

ma10<-capscale(pwiselrma~waters, add="lingoes")

print(ma10)

anova.cca(ma10,permutations=how(nperm=9999))

# ma11 - pairwise and roads

ma11<-capscale(pwiselrma~roads+Condition(dist), add="lingoes")

print(ma11)

anova.cca(ma11,permutations=how(nperm=9999))

# ma12 - pairwise and water

ma12<-capscale(pwiselrma~waters+Condition(dist), add="lingoes")

print(ma12)

anova.cca(ma12,permutations=how(nperm=9999))

# ma13 -pairwise and habitatGL1

ma13<-capscale(pwiselrma~habsg1+Condition(dist), add="lingoes")

print(ma13)

anova.cca(ma13,permutations=how(nperm=9999))

# ma14 -pairwise and habitatGL11

ma14<-capscale(pwiselrma~habsg11+Condition(dist), add="lingoes")

print(ma14)

anova.cca(ma14,permutations=how(nperm=9999))

# ma15 -pairwise and habitatGL31

ma15<-capscale(pwiselrma~habsg31+Condition(dist), add="lingoes")

print(ma15)

anova.cca(ma15,permutations=how(nperm=9999))

# ma16 -pairwise and habitatGL51

ma16<-capscale(pwiselrma~habsg51+Condition(dist), add="lingoes")

print(ma16)

anova.cca(ma16,permutations=how(nperm=9999))

# ma17 -pairwise and habitatGL71

ma17<-capscale(pwiselrma~habsg71+Condition(dist), add="lingoes")

print(ma17)

anova.cca(ma17,permutations=how(nperm=9999))

# ma18 -pairwise and habitatGL91

ma18<-capscale(pwiselrma~habsg91+Condition(dist), add="lingoes")

print(ma18)

anova.cca(ma18,permutations=how(nperm=9999))

# ma19 -pairwise and habitatGL99

ma19<-capscale(pwiselrma~habsg99+Condition(dist), add="lingoes")

print(ma19)

anova.cca(ma19,permutations=how(nperm=9999))

############################################################################

# run dbRDA models - one per explanatory variable

# set 2 - QGM pairwise models (mb)

# pairwise matrix as response variable - distance matrices created at start

# distance, habitats, roads, and water as explanatory variables - PCos matrices created above

# mb1 - pairwise and distance

mb1<-capscale(pwiseqgma~dist, add="lingoes")

print(mb1)

anova.cca(mb1,permutations=how(nperm=9999))

# mb2 - pairwise and habitatGL1

mb2<-capscale(pwiseqgma~habsg1, add="lingoes")

print(mb2)

anova.cca(mb2,permutations=how(nperm=9999))

# mb3 - pairwise and habitatGL11

mb3<-capscale(pwiseqgma~habsg11, add="lingoes")

print(mb3)

anova.cca(mb3,permutations=how(nperm=9999))

# mb4 - pairwise and habitatGL31

mb4<-capscale(pwiseqgma~habsg31, add="lingoes")

print(mb4)

anova.cca(mb4,permutations=how(nperm=9999))

# mb5 - pairwise and habitatGL51

mb5<-capscale(pwiseqgma~habsg51, add="lingoes")

print(mb5)

anova.cca(mb5,permutations=how(nperm=9999))

# mb6 - pairwise and habitatGL71

mb6<-capscale(pwiseqgma~habsg71, add="lingoes")

print(mb6)

anova.cca(mb6,permutations=how(nperm=9999))

# mb7 - pairwise and habitatGL91

mb7<-capscale(pwiseqgma~habsg91, add="lingoes")

print(mb7)

anova.cca(mb7,permutations=how(nperm=9999))

# mb8 - pairwise and habitatGL99

mb8<-capscale(pwiseqgma~habsg99, add="lingoes")

print(mb8)

anova.cca(mb8,permutations=how(nperm=9999))

# mb9 - pairwise and roads

mb9<-capscale(pwiseqgma~roads, add="lingoes")

print(mb9)

anova.cca(mb9,permutations=how(nperm=9999))

# mb10 - pairwise and water

mb10<-capscale(pwiseqgma~waters, add="lingoes")

print(mb10)

anova.cca(mb10,permutations=how(nperm=9999))

# mb11 - pairwise and roads

mb11<-capscale(pwiseqgma~roads+Condition(dist), add="lingoes")

print(mb11)

anova.cca(mb11,permutations=how(nperm=9999))

# mb12 - pairwise and water

mb12<-capscale(pwiseqgma~waters+Condition(dist), add="lingoes")

print(mb12)

anova.cca(mb12,permutations=how(nperm=9999))

# mb13 - pairwise and habitatGL1

mb13<-capscale(pwiseqgma~habsg1+Condition(dist), add="lingoes")

print(mb13)

anova.cca(mb13,permutations=how(nperm=9999))

# mb14 - pairwise and habitatGL11

mb14<-capscale(pwiseqgma~habsg11+Condition(dist), add="lingoes")

print(mb14)

anova.cca(mb14,permutations=how(nperm=9999))

# mb15 - pairwise and habitatGL31

mb15<-capscale(pwiseqgma~habsg31+Condition(dist), add="lingoes")

print(mb15)

anova.cca(mb15,permutations=how(nperm=9999))

# mb16 - pairwise and habitatGL51

mb16<-capscale(pwiseqgma~habsg51+Condition(dist), add="lingoes")

print(mb16)

anova.cca(mb16,permutations=how(nperm=9999))

# mb17 - pairwise and habitatGL71

mb17<-capscale(pwiseqgma~habsg71+Condition(dist), add="lingoes")

print(mb17)

anova.cca(mb17,permutations=how(nperm=9999))

# mb18 - pairwise and habitatGL91

mb18<-capscale(pwiseqgma~habsg91+Condition(dist), add="lingoes")

print(mb18)

anova.cca(mb18,permutations=how(nperm=9999))

# mb19 - pairwise and habitatGL99

mb19<-capscale(pwiseqgma~habsg99+Condition(dist), add="lingoes")

print(mb19)

anova.cca(mb19,permutations=how(nperm=9999))

############################################################################

# extract adjusted R2 for all models

RsquareAdj(ma1)

RsquareAdj(ma2)

RsquareAdj(ma3)

RsquareAdj(ma4)

RsquareAdj(ma5)

RsquareAdj(ma6)

RsquareAdj(ma7)

RsquareAdj(ma8)

RsquareAdj(ma9)

RsquareAdj(ma10)

RsquareAdj(ma11)

RsquareAdj(ma12)

RsquareAdj(ma13)

RsquareAdj(ma14)

RsquareAdj(ma15)

RsquareAdj(ma16)

RsquareAdj(ma17)

RsquareAdj(ma18)

RsquareAdj(ma19)

RsquareAdj(mb1)

RsquareAdj(mb2)

RsquareAdj(mb3)

RsquareAdj(mb4)

RsquareAdj(mb5)

RsquareAdj(mb6)

RsquareAdj(mb7)

RsquareAdj(mb8)

RsquareAdj(mb9)

RsquareAdj(mb10)

RsquareAdj(mb11)

RsquareAdj(mb12)

RsquareAdj(mb13)

RsquareAdj(mb14)

RsquareAdj(mb15)

RsquareAdj(mb16)

RsquareAdj(mb17)

RsquareAdj(mb18)

RsquareAdj(mb19)

############################################################################

# END
