# Supplementary material for: Evidence for High Levels of Gene Flow in Hedgehogs (Erinaceus europaeus) Across South Wales, UK, Despite Potential Anthropogenic and Natural Barriers to Dispersal
Source: Ecol Evol. 2025 Apr 7;15(4):e71201. doi: 10.1002/ece3.71201 (PMC11975622; doi:10.1002/ece3.71201)
Supplement: Supplementary file 2 — Data S2. [file ECE3-15-e71201-s002.docx]

**Appendix for Shove et al.**

Previously published studies used a variety of means to investigate species-habitat interactions or to define habitat resistance , varying from simple categories, e.g. (Pettett *et al.* 2017), or positive and negative associations, e.g. (Doncaster, Rondinini & Johnson 2001), to generate resistance values that were then tested across several models, e.g. (Driezen *et al.* 2007). These results were collated and summarised to produce a typical resistance level and resistance value which led to the GIS input value (the midpoint value) for mapping purposes.

**Table S1: Multiplex microsatellites used for hedgehog genotyping**

| **Multiplex** | **Locus** | **Allele Size (bp)** | **Modification** | **Primer sequence F** | **Primer sequence R** | **Reference** |
| --- | --- | --- | --- | --- | --- | --- |
| 1 | EEU12H | 91-97 | blue (6-FAM) | CTGCATGTACCTCTCCTCTACCTC | TTTTCTTTTTCCACCGGTGTTATC | Henderson *et al.* (2000) |
|  | EEU1 | 129-143 | green (HEX) | ACCCACATCTTATGCCTTTCAGTGAT | TAAATGTCAATGGCCATCTGTTATAACAA | Becher and Griffiths (1997) |
|  | EEU4 | 144-170 | blue (6-FAM) | GTGGTTAGAGCAGCAAGGACT | AGCTCTTAGCACTGGGTCTGA | Becher and Griffiths (1997) |
|  | EEU37H | 236-280 | green (HEX) | ATGAGGTGAGGCTTACCAAAAA | GGAATCTCACAGATGTAAAGTTCTAGC | Henderson *et al.* (2000) |
|  | E13 | 310 | blue (6-FAM) | tgaggacccaggttcaagtc | agtgtagggtgggcagaaaa | Curto *et al.* (2019) |
| 2 | EEU5 | 107-139 | green (HEX) | TGCATGAGGAACCAAATTCAA | CAGCATGGATGTCCCACTACT | Becher and Griffiths (1997) |
|  | EEU3 | 131-181 | blue (6-FAM) | TCAACAGAAGACAGGAGCAGATACAGG | GAACTTCCACCAGAACATCAAGGCT | Becher and Griffiths (1997) |
|  | EEU6 | 145-159 | green (HEX) | CAGTGAAGTTAAGGGTGGCTTT | TATGCTGGGTGGGTCTCTTCT | Becher and Griffiths (1997) |
|  | EEU2 | 257-281 | blue (6-FAM) | GTAGGGACCGAGGGCTTGAACTG | GACTGGCATTCACCCTAAAACACAT | Becher and Griffiths (1997) |
| 3 | W23 | 114-126 | blue (6-FAM) | agctactggatggatgggtg | atctcacttagcacgacccc | Curto *et al.* (2019) |
|  | EEU43H | 156-168 | green (HEX) | CCATGTACAGTGGATTTACCTGC | ACCCTAGGAGCAACTTGGAGAT | Henderson *et al.* (2000) |
|  | W30 | 177-197 | blue (6-FAM) | gagccaggtccccattgtat | agtccaaggcaaactcaggt | Curto *et al.* (2019) |
|  | W8 | 244-269 | blue (6-FAM) | ttccagcctcccatatgtca | ggtagtgggaactgtgtgca | Curto *et al.* (2019) |
|  | E36 | 319-348 | green (HEX) | gtgaagacagggaagcttgc | ggcttggtgggtgtactttg | Curto *et al.* (2019) |

**Table S2: Summary of habitat resistance values by Phase 1 habitat type. Shaded rows are based on estimates from comparable habitats; see Table S3 for details. Habitat codes and types are from the JNCC Phase I Habitat Survey handbook (JNCC 2010)**

| **Habitat Code** | **Habitat Type** | **Typical Resistance Level** | **Typical Resistance Value (0 to 99)** | **GIS input value** |
| --- | --- | --- | --- | --- |
| A.1.1.1 | Broadleaved semi-natural woodland | Low | 2 to 20 | 11 |
| A.1.1.2 | Broadleaved plantation | Low-medium | 21 to 40 | 31 |
| A.1.2.1 | Coniferous semi-natural woodland | Medium | 41 to 60 | 51 |
| A.1.2.2 | Coniferous plantation | Medium | 41 to 60 | 51 |
| A.1.3.1 | Mixed semi-natural woodland | Low-medium | 21 to 40 | 31 |
| A.1.3.2 | Mixed plantation | Low-medium | 21 to 40 | 31 |
| A.2.1 | Dense / continuous scrub | Negligible | 1 | 1 |
| A.2.2 | Scattered scrub | Low | 2 to 20 | 11 |
| A.3.1 | Broadleaved parkland / scattered trees | Negligible | 1 | 1 |
| A.3.2 | Coniferous parkland / scattered trees | Negligible | 1 | 1 |
| A.3.3 | Mixed parkland / scattered trees | Negligible | 1 | 1 |
| A.4.1 | Broadleaved recently felled woodland | Low | 2 to 20 | 11 |
| A.4.2 | Coniferous recently felled woodland | Low-medium | 21 to 40 | 31 |
| A.4.3 | Mixed recently felled woodland | Low-medium | 21 to 40 | 31 |
| B.1.1 | Unimproved acid grassland | Negligible | 1 | 1 |
| B.1.2 | Semi-improved acid grassland | Negligible | 1 | 1 |
| B.2.1 | Unimproved neutral grassland | Negligible | 1 | 1 |
| B.2.2 | Semi-improved neutral grassland | Negligible | 1 | 1 |
| B.3.1 | Unimproved calcareous grassland | Negligible | 1 | 1 |
| B.3.2 | Semi-improved calcareous grassland | Negligible | 1 | 1 |
| B.4 | Improved grassland (pasture) | Negligible | 1 | 1 |
| B.5 | Marsh / marshy grassland | Negligible | 1 | 1 |
| B.6 | Poor semi-improved grassland | Negligible | 1 | 1 |
| C.1.1 | Continuous bracken | Negligible | 1 | 1 |
| C.1.2 | Scattered bracken | Low-medium | 21 to 40 | 31 |
| C.2 | Upland species-rich ledges | High | 81 to 98 | 91 |
| C.3.1 | Tall ruderal | Negligible | 1 | 1 |
| C.3.2 | Non-ruderal | Negligible | 1 | 1 |
| D.1.1 | Dry dwarf shrub heath – acid | Low-medium | 21 to 40 | 31 |
| D.1.2 | Dry dwarf shrub heath – basic | Low-medium | 21 to 40 | 31 |
| D.2 | Wet dwarf shrub heath | Medium-high | 61 to 80 | 71 |
| D.3 | Lichen / bryophyte heath | Medium-high | 61 to 80 | 71 |
| D.4 | Montane heath / dwarf herb | Medium | 41 to 60 | 51 |
| D.5 | Dry heath / acid grassland mosaic | Low-medium | 21 to 40 | 31 |
| D.6 | Wet heath / acid grassland mosaic | Medium | 41 to 60 | 51 |
| E.1.6.1 | Blanket bog | High | 81 to 98 | 91 |
| E.1.6.2 | Raised bog | High | 81 to 98 | 91 |
| E.1.7 | Wet modified bog | High | 81 to 98 | 91 |
| E.1.8 | Dry modified bog | High | 81 to 98 | 91 |
| E.2.1 | Acid / neutral flush / spring | High | 81 to 98 | 91 |
| E.2.2 | Basic flush / spring | High | 81 to 98 | 91 |
| E.2.3 | Bryophyte dominated flush / spring | High | 81 to 98 | 91 |
| E.3.1 | Valley mire fen | High | 81 to 98 | 91 |
| E.3.2 | Basin mire fen | High | 81 to 98 | 91 |
| E.3.3 | Flood plain fen | High | 81 to 98 | 91 |
| E.4 | Bare peat | High | 81 to 98 | 91 |
| F.1 | Swamp | High | 81 to 98 | 91 |
| F.2.1 | Marginal vegetation | High | 81 to 98 | 91 |
| F.2.2 | Inundation vegetation | High | 81 to 98 | 91 |
| G.1 | Standing water | High | 81 to 98 | 91 |
| G.1.1 | Eutrophic | High | 81 to 98 | 91 |
| G.1.2 | Mesotrophic | High | 81 to 98 | 91 |
| G.1.3 | Oligotrophic | High | 81 to 98 | 91 |
| G.1.4 | Dystrophic | High | 81 to 98 | 91 |
| G.1.5 | Marl | High | 81 to 98 | 91 |
| G.1.6 | Brackish | High | 81 to 98 | 91 |
| G.2 | Running water | High | 81 to 98 | 91 |
| G.2.1 | Eutrophic | High | 81 to 98 | 91 |
| G.2.2 | Mesotrophic | High | 81 to 98 | 91 |
| G.2.3 | Oligotrophic | High | 81 to 98 | 91 |
| G.2.4 | Dystrophic | High | 81 to 98 | 91 |
| G.2.5 | Marl | High | 81 to 98 | 91 |
| G.2.6 | Brackish | High | 81 to 98 | 91 |
| H.1.1 | Intertidal mud / sand | High | 81 to 98 | 91 |
| H.1.2 | Intertidal shingles / cobbles | High | 81 to 98 | 91 |
| H.1.3 | Intertidal boulders / rocks | High | 81 to 98 | 91 |
| H.1.(1-2).1 | Zostera beds | High | 81 to 98 | 91 |
| H.1.(1-3).2 | Green algal beds | High | 81 to 98 | 91 |
| H.1.(1-3).3 | Brown algal beds | High | 81 to 98 | 91 |
| H.2.3 | Saltmarsh / dune interface | High | 81 to 98 | 91 |
| H.2.4 | Scattered saltmarsh plants | High | 81 to 98 | 91 |
| H.2.6 | Dense / continuous saltmarsh plants | High | 81 to 98 | 91 |
| H.3 | Shingle above high tide mark | High | 81 to 98 | 91 |
| H.4 | Boulders / rocks above high tide mark | High | 81 to 98 | 91 |
| H.5 | Strandline vegetation | High | 81 to 98 | 91 |
| H.6.4 | Sand dune – dune slack | Medium-high | 61 to 80 | 71 |
| H.6.5 | Sand dune – dune grassland | Medium-high | 61 to 80 | 71 |
| H.6.6 | Sand dune – dune heath | High | 81 to 98 | 91 |
| H.6.7 | Sand dune – dune scrub | Medium-high | 61 to 80 | 71 |
| H.6.8 | Sand dune – open dune | High | 81 to 98 | 91 |
| H.8.1 | Maritime cliff and slope – hard cliff | High | 81 to 98 | 91 |
| H.8.2 | Maritime cliff and slope – soft cliff | High | 81 to 98 | 91 |
| H.8.3 | Maritime cliff and slope – crevice / ledge vegetation | High | 81 to 98 | 91 |
| H.8.4 | Maritime cliff and slope – coastal grassland | Negligible | 1 | 1 |
| H.8.5 | Maritime cliff and slope – coastal heathland | Low-medium | 21 to 40 | 31 |
| I.1.1.1 | Natural inland cliff – acid / neutral | High | 81 to 98 | 91 |
| I.1.1.2 | Natural inland cliff – basic | High | 81 to 98 | 91 |
| I.1.2.1 | Natural scree – acid / neutral | High | 81 to 98 | 91 |
| I.1.2.2 | Natural scree – basic | High | 81 to 98 | 91 |
| I.1.3 | Natural limestone pavement | Medium-high | 61 to 80 | 71 |
| I.1.4.1 | Natural other exposure – acid / neutral | High | 81 to 98 | 91 |
| I.1.4.2 | Natural other exposure – basic | High | 81 to 98 | 91 |
| I.1.5 | Natural cave | High | 81 to 98 | 91 |
| I.2.1 | Artificial quarry | High | 81 to 98 | 91 |
| I.2.2 | Artificial spoil | High | 81 to 98 | 91 |
| I.2.3 | Artificial mine | High | 81 to 98 | 91 |
| I.2.4 | Artificial refuse tip | High | 81 to 98 | 91 |
| J.1.1 | Arable | Medium-high | 61 to 80 | 71 |
| J.1.2 | Amenity grassland | Negligible | 1 | 1 |
| J.1.3 | Ephemeral / short perennial | Negligible | 1 | 1 |
| J.1.4 | Introduced shrub (inc INNS) | Negligible | 1 | 1 |
| J.1.5 | Gardens | Low | 2 to 20 | 11 |
| J.2.1.1 | Intact species rich hedge | Negligible | 1 | 1 |
| J.2.1.2 | Intact species poor hedge | Negligible | 1 | 1 |
| J.2.2.1 | Defunct species rich hedge | Negligible | 1 | 1 |
| J.2.2.2 | Defunct species poor hedge | Negligible | 1 | 1 |
| J.2.3.1 | Species rich hedge with trees | Negligible | 1 | 1 |
| J.2.3.2 | Species poor hedge with trees | Negligible | 1 | 1 |
| J.2.4 | Fence | Medium | 41 to 60 | 51 |
| J.2.5 | Wall | High | 81 to 98 | 91 |
| J.2.6 | Dry ditch | Negligible | 1 | 1 |
| J.2.7 | Boundary removed | Negligible | 1 | 1 |
| J.2.8 | Earth bank | Negligible | 1 | 1 |
| J.3.4 | Caravan site | Medium-high | 61 to 80 | 71 |
| J.3.5 | Sea wall | Complete | 99 | 99 |
| J.3.6 | Buildings | Complete | 99 | 99 |
| J.3.7 | Track / road | Medium | 41 to 60 | 51 |
| J.3.7 | Road – including all unpaved and asphalt roads, and railroads. | Low | 2 to 20 | 31 |
| J.3.7 | Large Road – as above but over 4m wide | High | 81 to 98 | 91 |
| J.4 | Bare ground | Low-medium | 21 to 40 | 31 |
| J.5 | Other habitat | Low-medium | 21 to 40 | 31 |
| J.5 | Solar panel fields | Low | 2 to 20 | 11 |

**Table S3: Habitat resistance values by Phase 1 habitat type. Data were not directly available for 49 of the habitat types (shaded in the table), so comparable habitats were used as a proxy. The data and conclusions from published studies were contradictory for 14 of the habitat types where data were directly available, so the most common result was used in combination with species ecology to determine the most appropriate resistance level.**

| **Habitat Code** | **Habitat Type** | **Summary of References** | **References** | **Typical Resistance Level** | **Typical Resistance Value (0 to 99)** | **Notes** |
| --- | --- | --- | --- | --- | --- | --- |
| A.1.1.1 | Broadleaved semi-natural woodland | Positive link to edge habitats | (Huijser & Bergers 2000) | Low | 2 to 20 | Woodland habitats noted to have low hedgehog densities - boundaries used more |
|  |  | Positive association | (Doncaster, Rondinini & Johnson 2001) |  |  |  |
|  |  | Used but not as much as gardens and fields | (Rondinini & Doncaster 2002) |  |  |  |
|  |  | Extensive use, especially in clearings | (Riber 2006) |  |  |  |
|  |  | Second highest observation rate, negative link in greater extents, less so in edge habitats | (Hof & Bright 2012) |  |  |  |
|  |  | Positive effect - as linear habitats (edge effect) | (van de Poel, Dekker & Langevelde 2015) |  |  |  |
|  |  | Least or second least ranked | (Pettett *et al.* 2017) |  |  |  |
|  |  | Negative relationship | (Hof, Allen & Bright 2019) |  |  |  |
|  |  | Favourable habitat | (Wright *et al.* 2020) |  |  |  |
|  |  | Winter nesting - undergrowth needed | (Reeve 1981) |  |  |  |
|  |  | Neither selected for or against but higher than pasture/improved grassland, clearings and edge habitats important | (Hof 2009) |  |  |  |
|  |  | Positive preference in spring and summer, not so in autumn | (Dowie 1993) |  |  |  |
| A.1.1.2 | Broadleaved plantation | Positive link to edge habitats | (Huijser & Bergers 2000) | Low-medium | 21 to 40 | Woodland habitats noted to have low hedgehog densities - boundaries used more |
|  |  | Positive association | (Doncaster, Rondinini & Johnson 2001) |  |  |  |
|  |  | Used but not as much as gardens and fields | (Rondinini & Doncaster 2002) |  |  |  |
|  |  | Extensive use, especially in clearings | (Riber 2006) |  |  |  |
|  |  | Second highest observation rate, negative link in greater extents, less so in edge habitats | (Hof & Bright 2012) |  |  |  |
|  |  | Positive effect - as linear habitats (edge effect) | (van de Poel, Dekker & Langevelde 2015) |  |  |  |
|  |  | Least or second least ranked | (Pettett *et al.* 2017) |  |  |  |
|  |  | Negative relationship | (Hof, Allen & Bright 2019) |  |  |  |
|  |  | Favourable habitat | (Wright *et al.* 2020) |  |  |  |
|  |  | Winter nesting - undergrowth needed | (Reeve 1981) |  |  |  |
|  |  | Neither selected for or against but higher than pasture/improved grassland, clearings and edge habitats important | (Hof 2009) |  |  |  |
|  |  | Positive preference in spring and summer, not so in autumn | (Dowie 1993) |  |  |  |
| A.1.2.1 | Coniferous semi-natural woodland | Lowest observation rate, negative link in greater extents, less so in edge habitats | (Hof & Bright 2012) | Medium | 41 to 60 | Less well used than other woodland habitats, mixed results - edge habitat used more |
|  |  | Positive effect - as linear habitats (edge effect) | (van de Poel, Dekker & Langevelde 2015) |  |  |  |
|  |  | Least or second least ranked | (Pettett *et al.* 2017) |  |  |  |
|  |  | High selection | (Hof 2009) |  |  |  |
| A.1.2.2 | Coniferous plantation | Lowest observation rate, negative link in greater extents, less so in edge habitats | (Hof & Bright 2012) | Medium | 41 to 60 | Less well used than other woodland habitats, mixed results - edge habitat used more |
|  |  | Positive effect - as linear habitats (edge effect) | (van de Poel, Dekker & Langevelde 2015) |  |  |  |
|  |  | Least or second least ranked | (Pettett *et al.* 2017) |  |  |  |
|  |  | High selection | (Hof 2009) |  |  |  |
| A.1.3.1 | Mixed semi-natural woodland | Highly selected (1 to 5m within habitat) | (Hof & Bright 2010) | Low-medium | 21 to 40 | Woodland habitats noted to have low hedgehog densities - boundaries used more |
|  |  | Negative link in greater extents, less so in edge habitats | (Hof & Bright 2012) |  |  |  |
|  |  | Positive effect - as linear habitats (edge effect) | (van de Poel, Dekker & Langevelde 2015) |  |  |  |
|  |  | Least or second least ranked | (Pettett *et al.* 2017) |  |  |  |
|  |  | Neither selected for or against but higher than pasture/improved grassland, clearings and edge habitats important | (Hof 2009) |  |  |  |
| A.1.3.2 | Mixed plantation | Highly selected (1 to 5m within habitat) | (Hof & Bright 2010) | Low-medium | 21 to 40 | Woodland habitats noted to have low hedgehog densities - boundaries used more |
|  |  | Negative link in greater extents, less so in edge habitats | (Hof & Bright 2012) |  |  |  |
|  |  | Positive effect - as linear habitats (edge effect) | (van de Poel, Dekker & Langevelde 2015) |  |  |  |
|  |  | Least or second least ranked | (Pettett *et al.* 2017) |  |  |  |
|  |  | Neither selected for or against but higher than pasture/improved grassland, clearings and edge habitats important | (Hof 2009) |  |  |  |
| A.2.1 | Dense / continuous scrub | Used for cover | (Rondinini & Doncaster 2002) | Negligible | 1 | Considered similar to hedgerows? Could depend on extent |
|  |  | Positive effect - as linear habitats (edge effect) | (van de Poel, Dekker & Langevelde 2015) |  |  |  |
|  |  | Confirmed habitat use | (Berger *et al.* 2020a) |  |  |  |
|  |  | Positive link | (Yarnell & Pettett 2020) |  |  |  |
|  |  | Confirmed use | (Berger *et al.* 2020b) |  |  |  |
|  |  | Winter nesting | (Reeve 1981) |  |  |  |
| A.2.2 | Scattered scrub | Confirmed habitat use | (Berger *et al.* 2020a) | Low | 2 to 20 | Limited cover but may be used in similar manner to less structured gardens / open habitats, activity focused to edges |
| A.3.1 | Broadleaved parkland / scattered trees | Positive link | (Hof & Bright 2009) | Negligible | 1 | Hedgehogs reported to use parkland areas |
|  |  | Confirmed habitat use | (Berger *et al.* 2020a) |  |  |  |
|  |  | Confirmed use | (Berger *et al.* 2020b) |  |  |  |
| A.3.2 | Coniferous parkland / scattered trees | Positive link | (Hof & Bright 2009) | Negligible | 1 | Hedgehogs reported to use parkland areas |
|  |  | Confirmed habitat use | (Berger *et al.* 2020a) |  |  |  |
|  |  | Confirmed use | (Berger *et al.* 2020b) |  |  |  |
| A.3.3 | Mixed parkland / scattered trees | Positive link | (Hof & Bright 2009) | Negligible | 1 | Hedgehogs reported to use parkland areas |
|  |  | Confirmed habitat use | (Berger *et al.* 2020a) |  |  |  |
|  |  | Confirmed use | (Berger *et al.* 2020b) |  |  |  |
| A.4.1 | Broadleaved recently felled woodland | No references | | Low | 2 to 20 | Open areas but hedgehogs known to use parkland and open spaces, may be limited to edge habitats  Proxy = broadleaved woodland and parkland |
| A.4.2 | Coniferous recently felled woodland | No references | | Low-medium | 21 to 40 | Open areas but hedgehogs known to use parkland and open spaces, may be limited to edge habitats  Proxy = broadleaved woodland and parkland |
| A.4.3 | Mixed recently felled woodland | No references | | Low-medium | 21 to 40 | Open areas but hedgehogs known to use parkland and open spaces, may be limited to edge habitats  Proxy = broadleaved woodland and parkland |
| B.1.1 | Unimproved acid grassland | Preferred habitat | (Micol, Doncaster & Mackinlay 1994) | Negligible | 1 | Pasture and set aside well used |
|  |  | Positive association | (Doncaster, Rondinini & Johnson 2001) |  |  |  |
|  |  | Scarce in pasture | (Young *et al.* 2006) |  |  |  |
|  |  | Highly selected (margins) | (Hof & Bright 2010) |  |  |  |
|  |  | Second highest observation rate | (Hof & Bright 2012) |  |  |  |
|  |  | Margins used but less impact on movement distance | (Moorhouse *et al.* 2014) |  |  |  |
|  |  | Middle ranking - changeable | (Pettett *et al.* 2017) |  |  |  |
|  |  | Present | (Williams *et al.* 2018) |  |  |  |
|  |  | Positive link (field margins) | (Yarnell & Pettett 2020) |  |  |  |
|  |  | Less preferred | (Braaker *et al.* 2014) |  |  |  |
|  |  | Favourable habitat | (Wright *et al.* 2020) |  |  |  |
|  |  | Summer nesting in taller vegetation | (Reeve 1981) |  |  |  |
|  |  | Positive correlation | (Hof 2009) |  |  |  |
|  |  | Less preference unless used as pasture | (Dowie 1993) |  |  |  |
| B.1.2 | Semi-improved acid grassland | Preferred habitat | (Micol, Doncaster & Mackinlay 1994) | Negligible | 1 | Pasture and set aside well used |
|  |  | Positive association | (Doncaster, Rondinini & Johnson 2001) |  |  |  |
|  |  | Scarce in pasture | (Young *et al.* 2006) |  |  |  |
|  |  | Highly selected (margins) | (Hof & Bright 2010) |  |  |  |
|  |  | Second highest observation rate | (Hof & Bright 2012) |  |  |  |
|  |  | Margins used but less imapct on movement distance | (Moorhouse *et al.* 2014) |  |  |  |
|  |  | Middle ranking - changeable | (Pettett *et al.* 2017) |  |  |  |
|  |  | Present | (Williams *et al.* 2018) |  |  |  |
|  |  | Positive link (field margins) | (Yarnell & Pettett 2020) |  |  |  |
|  |  | Less preferred | (Braaker *et al.* 2014) |  |  |  |
|  |  | Favourable habitat | (Wright *et al.* 2020) |  |  |  |
|  |  | Summer nesting in taller vegetation | (Reeve 1981) |  |  |  |
|  |  | Positive correlation | (Hof 2009) |  |  |  |
|  |  | Less preference unless used as pasture | (Dowie 1993) |  |  |  |
| B.2.1 | Unimproved neutral grassland | Preferred habitat | (Micol, Doncaster & Mackinlay 1994) | Negligible | 1 | Pasture and set aside well used |
|  |  | Positive association | (Doncaster, Rondinini & Johnson 2001) |  |  |  |
|  |  | Scarce in pasture | (Young *et al.* 2006) |  |  |  |
|  |  | Highly selected (margins) | (Hof & Bright 2010) |  |  |  |
|  |  | Second highest observation rate | (Hof & Bright 2012) |  |  |  |
|  |  | Margins used but less imapct on movement distance | (Moorhouse *et al.* 2014) |  |  |  |
|  |  | Middle ranking - changeable | (Pettett *et al.* 2017) |  |  |  |
|  |  | Present | (Williams *et al.* 2018) |  |  |  |
|  |  | Positive link (field margins) | (Yarnell & Pettett 2020) |  |  |  |
|  |  | Less preferred | (Braaker *et al.* 2014) |  |  |  |
|  |  | Favourable habitat | (Wright *et al.* 2020) |  |  |  |
|  |  | Summer nesting in taller vegetation | (Reeve 1981) |  |  |  |
|  |  | Neither selected for or against but higher than pasture/improved grassland | (Hof 2009) |  |  |  |
|  |  | Positive preference | (Dowie 1993) |  |  |  |
| B.2.2 | Semi-improved neutral grassland | Preferred habitat | (Micol, Doncaster & Mackinlay 1994) | Negligible | 1 | Pasture and set aside well used |
|  |  | Positive association | (Doncaster, Rondinini & Johnson 2001) |  |  |  |
|  |  | Scarce in pasture | (Young *et al.* 2006) |  |  |  |
|  |  | Highly selected (margins) | (Hof & Bright 2010) |  |  |  |
|  |  | Second highest observation rate | (Hof & Bright 2012) |  |  |  |
|  |  | Margins used but less imapct on movement distance | (Moorhouse *et al.* 2014) |  |  |  |
|  |  | Middle ranking - changeable | (Pettett *et al.* 2017) |  |  |  |
|  |  | Present | (Williams *et al.* 2018) |  |  |  |
|  |  | Positive link (field margins) | (Yarnell & Pettett 2020) |  |  |  |
|  |  | Less preferred | (Braaker *et al.* 2014) |  |  |  |
|  |  | Favourable habitat | (Wright *et al.* 2020) |  |  |  |
|  |  | Summer nesting in taller vegetation | (Reeve 1981) |  |  |  |
|  |  | Neither selected for or against but higher than pasture/improved grassland | (Hof 2009) |  |  |  |
|  |  | Positive preference | (Dowie 1993) |  |  |  |
| B.3.1 | Unimproved calcareous grassland | Preferred habitat | (Micol, Doncaster & Mackinlay 1994) | Negligible | 1 | Pasture and set aside well used |
|  |  | Positive association | (Doncaster, Rondinini & Johnson 2001) |  |  |  |
|  |  | Scarce in pasture | (Young *et al.* 2006) |  |  |  |
|  |  | Highly selected (margins) | (Hof & Bright 2010) |  |  |  |
|  |  | Second highest observation rate | (Hof & Bright 2012) |  |  |  |
|  |  | Margins used but less imapct on movement distance | (Moorhouse *et al.* 2014) |  |  |  |
|  |  | Middle ranking - changeable | (Pettett *et al.* 2017) |  |  |  |
|  |  | Present | (Williams *et al.* 2018) |  |  |  |
|  |  | Positive link (field margins) | (Yarnell & Pettett 2020) |  |  |  |
|  |  | Less preferred | (Braaker *et al.* 2014) |  |  |  |
|  |  | Favourable habitat | (Wright *et al.* 2020) |  |  |  |
|  |  | Summer nesting in taller vegetation | (Reeve 1981) |  |  |  |
|  |  | Positive correlation, lower nutrients = less inverts = less food = fewer hedgehogs | (Hof 2009) |  |  |  |
|  |  | Less preference unless used as pasture | (Dowie 1993) |  |  |  |
| B.3.2 | Semi-improved calcareous grassland | Preferred habitat | (Micol, Doncaster & Mackinlay 1994) | Negligible | 1 | Pasture and set aside well used |
|  |  | Positive association | (Doncaster, Rondinini & Johnson 2001) |  |  |  |
|  |  | Scarce in pasture | (Young *et al.* 2006) |  |  |  |
|  |  | Highly selected (margins) | (Hof & Bright 2010) |  |  |  |
|  |  | Second highest observation rate | (Hof & Bright 2012) |  |  |  |
|  |  | Margins used but less imapct on movement distance | (Moorhouse *et al.* 2014) |  |  |  |
|  |  | Middle ranking - changeable | (Pettett *et al.* 2017) |  |  |  |
|  |  | Present | (Williams *et al.* 2018) |  |  |  |
|  |  | Positive link (field margins) | (Yarnell & Pettett 2020) |  |  |  |
|  |  | Less preferred | (Braaker *et al.* 2014) |  |  |  |
|  |  | Favourable habitat | (Wright *et al.* 2020) |  |  |  |
|  |  | Summer nesting in taller vegetation | (Reeve 1981) |  |  |  |
|  |  | Positive correlation, lower nutrients = less inverts = less food = fewer hedgehogs | (Hof 2009) |  |  |  |
|  |  | Less preference unless used as pasture | (Dowie 1993) |  |  |  |
| B.4 | Improved grassland (pasture) | Preferred habitat | (Micol, Doncaster & Mackinlay 1994) | Negligible | 1 | Pasture and set aside well used |
|  |  | Positive association | (Doncaster, Rondinini & Johnson 2001) |  |  |  |
|  |  | Scarce in pasture | (Young *et al.* 2006) |  |  |  |
|  |  | Highly selected (margins) | (Hof & Bright 2010) |  |  |  |
|  |  | Second highest observation rate | (Hof & Bright 2012) |  |  |  |
|  |  | Middle ranking - changeable | (Pettett *et al.* 2017) |  |  |  |
|  |  | Present | (Williams *et al.* 2018) |  |  |  |
|  |  | Negative relationship | (Hof, Allen & Bright 2019) |  |  |  |
|  |  | Positive link (field margins) | (Yarnell & Pettett 2020) |  |  |  |
|  |  | Less preferred | (Braaker *et al.* 2014) |  |  |  |
|  |  | Favourable habitat | (Wright *et al.* 2020) |  |  |  |
|  |  | Summer nesting in taller vegetation | (Reeve 1981) |  |  |  |
|  |  | Neither selected for or against | (Hof 2009) |  |  |  |
|  |  | Positive preference | (Dowie 1993) |  |  |  |
| B.5 | Marsh / marshy grassland | Seldom used | (Jackson 2007) | Negligible | 1 | Pasture and set aside well used |
|  |  | Highly selected (margins) | (Hof & Bright 2010) |  |  |  |
|  |  | Negative effect | (van de Poel, Dekker & Langevelde 2015) |  |  |  |
| B.6 | Poor semi-improved grassland | Preferred habitat | (Micol, Doncaster & Mackinlay 1994) | Negligible | 1 | Pasture and set aside well used |
|  |  | Positive association | (Doncaster, Rondinini & Johnson 2001) |  |  |  |
|  |  | Scarce in pasture | (Young *et al.* 2006) |  |  |  |
|  |  | Highly selected (margins) | (Hof & Bright 2010) |  |  |  |
|  |  | Second highest observation rate | (Hof & Bright 2012) |  |  |  |
|  |  | Margins used but less imapct on movement distance | (Moorhouse *et al.* 2014) |  |  |  |
|  |  | Middle ranking - changeable | (Pettett *et al.* 2017) |  |  |  |
|  |  | Present | (Williams *et al.* 2018) |  |  |  |
|  |  | Positive link (field margins) | (Yarnell & Pettett 2020) |  |  |  |
|  |  | Less preferred | (Braaker *et al.* 2014) |  |  |  |
|  |  | Favourable habitat | (Wright *et al.* 2020) |  |  |  |
|  |  | Summer nesting in taller vegetation | (Reeve 1981) |  |  |  |
|  |  | Neither selected for or against | (Hof 2009) |  |  |  |
|  |  | Less preference unless used as pasture | (Dowie 1993) |  |  |  |
| C.1.1 | Continuous bracken | Summer nesting in taller vegetation | (Reeve 1981) | Negligible | 1 | Similar to dense scrub in terms of cover provided |
| C.1.2 | Scattered bracken | No references | | Low-medium | 21 to 40 | Limited cover but may be used in similar manner to less structured gardens / open habitats, activity focused to edges  Proxy other habitat |
| C.2 | Upland species-rich ledges | No references | | High | 81 to 98 | Unlikely in upland areas, absent above treeline (Williams *et al.* 2018) |
| C.3.1 | Tall ruderal | Highly selected (margins) | (Hof & Bright 2010) | Negligible | 1 | Similar to dense scrub in terms of cover provided |
|  |  | Positive link (vegetation cover) | (Yarnell & Pettett 2020) |  |  |  |
| C.3.2 | Non-ruderal | No references | | Negligible | 1 | Similar to dense scrub in terms of cover provided  Proxy dense scrub |
| D.1.1 | Dry dwarf shrub heath - acid | Seldom used | (Jackson 2007) | Low-medium | 21 to 40 | Research suggests not well used but not necessarily a barrier |
|  |  | Lowest observation rate | (Hof & Bright 2012) |  |  |  |
| D.1.2 | Dry dwarf shrub heath - basic | Seldom used | (Jackson 2007) | Low-medium | 21 to 40 | Research suggests not well used but not necessarily a barrier |
|  |  | Lowest observation rate | (Hof & Bright 2012) |  |  |  |
| D.2 | Wet dwarf shrub heath | Seldom used | (Jackson 2007) | Medium-high | 61 to 80 | Research suggests not well used but not necessarily a barrier, wetter habitats noted to be used less frequently than dry habitats |
|  |  | Lowest observation rate | (Hof & Bright 2012) |  |  |  |
| D.3 | Lichen / bryophyte heath | Seldom used | (Jackson 2007) | Medium-high | 61 to 80 | Research suggests not well used but not necessarily a barrier, wetter habitats noted to be used less frequently than dry habitats |
|  |  | Lowest observation rate | (Hof & Bright 2012) |  |  |  |
| D.4 | Montane heath / dwarf herb | Seldom used | (Jackson 2007) | Medium | 41 to 60 | Research suggests not well used but not necessarily a barrier, upland habitats noted to be used in some studies |
|  |  | Lowest observation rate | (Hof & Bright 2012) |  |  |  |
| D.5 | Dry heath / acid grassland mosaic | No references | | Low-medium | 21 to 40 | Research suggests not well used but not necessarily a barrier  Proxy dry dwarf shrub heath - acid |
| D.6 | Wet heath / acid grassland mosaic | No references | | Medium | 41 to 60 | Research suggests not well used but not necessarily a barrier, wetter habitats noted to be used less frequently than dry habitats  Proxy wet dwarf shrub heath and acid grassland |
| E.1.6.1 | Blanket bog | Negative effect | (van de Poel, Dekker & Langevelde 2015) | High | 81 to 98 | Habitat type unlikely to be used |
| E.1.6.2 | Raised bog | Negative effect | (van de Poel, Dekker & Langevelde 2015) | High | 81 to 98 | Habitat type unlikely to be used |
| E.1.7 | Wet modified bog | Negative effect | (van de Poel, Dekker & Langevelde 2015) | High | 81 to 98 | Habitat type unlikely to be used |
| E.1.8 | Dry modified bog | Negative effect | (van de Poel, Dekker & Langevelde 2015) | High | 81 to 98 | Habitat type unlikely to be used |
| E.2.1 | Acid / neutral flush / spring | Negative effect | (van de Poel, Dekker & Langevelde 2015) | High | 81 to 98 | Habitat type unlikely to be used |
| E.2.2 | Basic flush / spring | Negative effect | (van de Poel, Dekker & Langevelde 2015) | High | 81 to 98 | Habitat type unlikely to be used |
| E.2.3 | Bryophyte dominated flush / spring | Negative effect | (van de Poel, Dekker & Langevelde 2015) | High | 81 to 98 | Habitat type unlikely to be used |
| E.3.1 | Valley mire fen | Negative effect | (van de Poel, Dekker & Langevelde 2015) | High | 81 to 98 | Habitat type unlikely to be used |
| E.3.2 | Basin mire fen | Negative effect | (van de Poel, Dekker & Langevelde 2015) | High | 81 to 98 | Habitat type unlikely to be used |
| E.3.3 | Flood plain fen | Negative effect | (van de Poel, Dekker & Langevelde 2015) | High | 81 to 98 | Habitat type unlikely to be used |
| E.4 | Bare peat | Negative effect | (van de Poel, Dekker & Langevelde 2015) | High | 81 to 98 | Habitat type unlikely to be used |
| F.1 | Swamp | Negative effect | (van de Poel, Dekker & Langevelde 2015) | High | 81 to 98 | Habitat type unlikely to be used |
| F.2.1 | Marginal vegetation | Negative effect | (van de Poel, Dekker & Langevelde 2015) | High | 81 to 98 | Habitat type unlikely to be used |
| F.2.2 | Inundation vegetation | Negative effect | (van de Poel, Dekker & Langevelde 2015) | High | 81 to 98 | Habitat type unlikely to be used |
| G.1 | Standing water | Partial barrier | (Hof & Bright 2009) | High | 81 to 98 | Hedgehogs known to swim on occasion so not an absolute barrier |
|  |  | Positive but not significant effect on presence | (Hof 2009) |  |  |  |
| G.1.1 | Eutrophic | Partial barrier | (Hof & Bright 2009) | High | 81 to 98 | Hedgehogs known to swim on occasion so not an absolute barrier |
|  |  | Positive but not significant effect on presence | (Hof 2009) |  |  |  |
| G.1.2 | Mesotrophic | Partial barrier | (Hof & Bright 2009) | High | 81 to 98 | Hedgehogs known to swim on occasion so not an absolute barrier |
|  |  | Positive but not significant effect on presence | (Hof 2009) |  |  |  |
| G.1.3 | Oligotrophic | Partial barrier | (Hof & Bright 2009) | High | 81 to 98 | Hedgehogs known to swim on occasion so not an absolute barrier |
|  |  | Positive but not significant effect on presence | (Hof 2009) |  |  |  |
| G.1.4 | Dystrophic | Partial barrier | (Hof & Bright 2009) | High | 81 to 98 | Hedgehogs known to swim on occasion so not an absolute barrier |
|  |  | Positive but not significant effect on presence | (Hof 2009) |  |  |  |
| G.1.5 | Marl | Partial barrier | (Hof & Bright 2009) | High | 81 to 98 | Hedgehogs known to swim on occasion so not an absolute barrier |
|  |  | Positive but not significant effect on presence | (Hof 2009) |  |  |  |
| G.1.6 | Brackish | Partial barrier | (Hof & Bright 2009) | High | 81 to 98 | Hedgehogs known to swim on occasion so not an absolute barrier |
|  |  | Positive but not significant effect on presence | (Hof 2009) |  |  |  |
| G.2 | Running water | Partial barrier | (Hof & Bright 2009) | High | 81 to 98 | Hedgehogs known to swim on occasion so not an absolute barrier |
|  |  | Negative effect on presence | (Hof 2009) |  |  |  |
| G.2.1 | Eutrophic | Partial barrier | (Hof & Bright 2009) | High | 81 to 98 | Hedgehogs known to swim on occasion so not an absolute barrier |
|  |  | Negative effect on presence | (Hof 2009) |  |  |  |
| G.2.2 | Mesotrophic | Partial barrier | (Hof & Bright 2009) | High | 81 to 98 | Hedgehogs known to swim on occasion so not an absolute barrier |
|  |  | Negative effect on presence | (Hof 2009) |  |  |  |
| G.2.3 | Oligotrophic | Partial barrier | (Hof & Bright 2009) | High | 81 to 98 | Hedgehogs known to swim on occasion so not an absolute barrier |
|  |  | Negative effect on presence | (Hof 2009) |  |  |  |
| G.2.4 | Dystrophic | Partial barrier | (Hof & Bright 2009) | High | 81 to 98 | Hedgehogs known to swim on occasion so not an absolute barrier |
|  |  | Negative effect on presence | (Hof 2009) |  |  |  |
| G.2.5 | Marl | Partial barrier | (Hof & Bright 2009) | High | 81 to 98 | Hedgehogs known to swim on occasion so not an absolute barrier |
|  |  | Negative effect on presence | (Hof 2009) |  |  |  |
| G.2.6 | Brackish | Partial barrier | (Hof & Bright 2009) | High | 81 to 98 | Hedgehogs known to swim on occasion so not an absolute barrier |
|  |  | Negative effect on presence | (Hof 2009) |  |  |  |
| H.1.1 | Intertidal mud / sand | No references | | High | 81 to 98 | Habitat type unlikely to be used  Proxy sand dune - open |
| H.1.2 | Intertidal shingles / cobbles | No references | | High | 81 to 98 | Habitat type unlikely to be usedProxy sand dune - open |
| H.1.3 | Intertidal boulders / rocks | No references | | High | 81 to 98 | Habitat type unlikely to be used  Proxy sand dune - open |
| H.1.(1-2).1 | Zostera beds | No references | | High | 81 to 98 | Habitat type unlikely to be used  Proxy sand dune - open |
| H.1.(1-3).2 | Green algal beds | No references | | High | 81 to 98 | Habitat type unlikely to be used  Proxy sand dune - open |
| H.1.(1-3).3 | Brown algal beds | No references | | High | 81 to 98 | Habitat type unlikely to be used  Proxy sand dune - open |
| H.2.3 | Saltmarsh / dune interface | No references | | High | 81 to 98 | Habitat type unlikely to be used  Proxy sand dune - open |
| H.2.4 | Scattered saltmarsh plants | No references | | High | 81 to 98 | Habitat type unlikely to be used  Proxy sand dune - open |
| H.2.6 | Dense / continuous saltmarsh plants | No references | | High | 81 to 98 | Habitat type unlikely to be used  Proxy sand dune - open |
| H.3 | Shingle above high tide mark | No references | | High | 81 to 98 | Habitat type unlikely to be used  Proxy sand dune - open |
| H.4 | Boulders / rocks above high tide mark | No references | | High | 81 to 98 | Habitat type unlikely to be used  Proxy sand dune - open |
| H.5 | Strandline vegetation | No references | | High | 81 to 98 | Habitat type unlikely to be used  Proxy sand dune - open |
| H.6.4 | Sand dune - dune slack | Foraging | (Jackson 2007) | Medium-high | 61 to 80 | Low use generally, only 1 study shows use, more due to proximity and presence within home range |
| H.6.5 | Sand dune - dune grassland | Foraging | (Jackson 2007) | Medium-high | 61 to 80 | Low use generally, only 1 study shows use, more due to proximity and presence within home range |
| H.6.6 | Sand dune - dune heath | No references | | High | 81 to 98 | Habitat type unlikely to be used  Proxy sand dune - open |
| H.6.7 | Sand dune - dune scrub | Foraging | (Jackson 2007) | Medium-high | 61 to 80 | Low use generally, only 1 study shows use, more due to proximity and presence within home range |
| H.6.8 | Sand dune - open dune | Not used | (Jackson 2007) | High | 81 to 98 | Not used |
| H.8.1 | Maritime cliff and slope - hard cliff | No references | | High | 81 to 98 | Habitat type unlikely to be used |
| H.8.2 | Maritime cliff and slope - soft cliff | No references | | High | 81 to 98 | Habitat type unlikely to be used |
| H.8.3 | Maritime cliff and slope - crevice / ledge vegetation | No references | | High | 81 to 98 | Habitat type unlikely to be used |
| H.8.4 | Maritime cliff and slope - coastal grassland | No references | | Negligible | 1 | Similar to grassland habitats in terms of potential use |
| H.8.5 | Maritime cliff and slope - coastal heathland | No references | | Low-medium | 21 to 40 | Heathland habitats generally not used |
| I.1.1.1 | Natural inland cliff - acid / neutral | No references | | High | 81 to 98 | Habitat type unlikely to be used |
| I.1.1.2 | Natural inland cliff - basic | No references | | High | 81 to 98 | Habitat type unlikely to be used |
| I.1.2.1 | Natural scree - acid / neutral | No references | | High | 81 to 98 | Habitat type unlikely to be used |
| I.1.2.2 | Natural scree - basic | No references | | High | 81 to 98 | Habitat type unlikely to be used |
| I.1.3 | Natural limestone pavement | No references | | Medium-high | 61 to 80 | Might vary depending on associated habitats |
| I.1.4.1 | Natural other exposure - acid / neutral | No references | | High | 81 to 98 | Habitat type unlikely to be used |
| I.1.4.2 | Natural other exposure - basic | No references | | High | 81 to 98 | Habitat type unlikely to be used |
| I.1.5 | Natural cave | No references | | High | 81 to 98 | Habitat type unlikely to be used |
| I.2.1 | Artificial quarry | No references | | High | 81 to 98 | Habitat type unlikely to be used |
| I.2.2 | Artificial spoil | No references | | High | 81 to 98 | Habitat type unlikely to be used |
| I.2.3 | Artificial mine | No references | | High | 81 to 98 | Habitat type unlikely to be used |
| I.2.4 | Artificial refuse tip | No references | | High | 81 to 98 | Habitat type unlikely to be used |
| J.1.1 | Arable | Negative link | (Micol, Doncaster & Mackinlay 1994) | Medium-high | 61 to 80 | Depends on size of fields but research suggests habitat is not used |
|  |  | Negative association | (Doncaster, Rondinini & Johnson 2001) |  |  |  |
|  |  | Rarely visited | (Riber 2006) |  |  |  |
|  |  | Rarely selected | (Hof & Bright 2010) |  |  |  |
|  |  | Positive effect | (van de Poel, Dekker & Langevelde 2015) |  |  |  |
|  |  | Least or second least ranked | (Pettett *et al.* 2017) |  |  |  |
|  |  | Present | (Williams *et al.* 2018) |  |  |  |
|  |  | Positive relationship | (Hof, Allen & Bright 2019) |  |  |  |
|  |  | Positive link (field margins) | (Yarnell & Pettett 2020) |  |  |  |
|  |  | Trended to avoid | (Driezen *et al.* 2007) |  |  |  |
|  |  | Favourable habitat | (Wright *et al.* 2020) |  |  |  |
|  |  | Selected against - use of field margins and hedgerows confuses this (use generally within 5m of edge) | (Hof 2009) |  |  |  |
|  |  | Negatively preferred | (Dowie 1993) |  |  |  |
| J.1.2 | Amenity grassland | Preferred habitat | (Micol, Doncaster & Mackinlay 1994) | Negligible | 1 | Amenity grassland noted to be well used |
|  |  | Positive association | (Doncaster, Rondinini & Johnson 2001) |  |  |  |
|  |  | Preferred | (Rondinini & Doncaster 2002) |  |  |  |
|  |  | Lower densities | (Orłowski & Nowak 2004) |  |  |  |
|  |  | More abundant | (Young *et al.* 2006) |  |  |  |
|  |  | Positive link | (Hof & Bright 2009) |  |  |  |
|  |  | Highly selected (females) | (Hof & Bright 2010) |  |  |  |
|  |  | Positive effect | (van de Poel, Dekker & Langevelde 2015) |  |  |  |
|  |  | Locally high, landscape lower | (Pettett *et al.* 2017) |  |  |  |
|  |  | Present | (Williams *et al.* 2018) |  |  |  |
|  |  | Confirmed habitat use | (Berger *et al.* 2020a) |  |  |  |
|  |  | Positive link | (Yarnell & Pettett 2020) |  |  |  |
|  |  | Strong preference, particularly with structures (bushes, trees etc) | (Braaker *et al.* 2014) |  |  |  |
|  |  | Favourable habitat | (Wright *et al.* 2020) |  |  |  |
|  |  | General use | (Reeve 1981) |  |  |  |
|  |  | Selected for - edge habitats | (Hof 2009) |  |  |  |
| J.1.3 | Ephemeral / short perennial | Positive link (vegetation cover) | (Yarnell & Pettett 2020) | Negligible | 1 | Similar to dense scrub, although less cover provided |
|  |  | Used | (Hof 2009) |  |  |  |
| J.1.4 | Introduced shrub (inc INNS) | No references | | Negligible | 1 | Similar to dense scrubProxy dense scrub |
| J.1.5 | Gardens | Preferred habitat | (Micol, Doncaster & Mackinlay 1994) | Low | 2 to 20 | Boundaries will affect resistance level |
|  |  | Positive association | (Doncaster, Rondinini & Johnson 2001) |  |  |  |
|  |  | Preferred | (Rondinini & Doncaster 2002) |  |  |  |
|  |  | Higher densities | (Orłowski & Nowak 2004) |  |  |  |
|  |  | More abundant | (Young *et al.* 2006) |  |  |  |
|  |  | High % of shrubs and grass | (Hof & Bright 2009) |  |  |  |
|  |  | Strong preference | (Dowding *et al.* 2010) |  |  |  |
|  |  | Highly selected (females) | (Hof & Bright 2010) |  |  |  |
|  |  | Highest observation rate | (Hof & Bright 2012) |  |  |  |
|  |  | Positive effect | (van de Poel, Dekker & Langevelde 2015) |  |  |  |
|  |  | Well used - likely under-recorded | (Williams, Stafford & Goodenough 2015) |  |  |  |
|  |  | Highest ranked | (Pettett *et al.* 2017) |  |  |  |
|  |  | Present | (Williams *et al.* 2018) |  |  |  |
|  |  | Positive link | (Yarnell & Pettett 2020) |  |  |  |
|  |  | Confirmed use | (Berger *et al.* 2020b) |  |  |  |
|  |  | Favoured | (Driezen *et al.* 2007) |  |  |  |
|  |  | Strong preference, particularly with structures (bushes, trees etc) | (Braaker *et al.* 2014) |  |  |  |
|  |  | Favourable habitat | (Wright *et al.* 2020) |  |  |  |
|  |  | General use but less than amenity/golf course | (Reeve 1981) |  |  |  |
|  |  | Used but limited by connectivity (or lack of) | (Hof 2009) |  |  |  |
|  |  | Mixed levels of use | (Dowie 1993) |  |  |  |
|  |  | Consistently preferred | (Dowding 2007) |  |  |  |
| J.2.1.1 | Intact species rich hedge | Positive link | (Micol, Doncaster & Mackinlay 1994) | Negligible | 1 |  |
|  |  | Positive link to edge habitats | (Huijser & Bergers 2000) |  |  |  |
|  |  | Positive association | (Doncaster, Rondinini & Johnson 2001) |  |  |  |
|  |  | Favoured (edge habitats) | (Riber 2006) |  |  |  |
|  |  | Highly selected | (Hof & Bright 2010) |  |  |  |
|  |  | Positive association | (Hof & Bright 2012) |  |  |  |
|  |  | Positive effect | (van de Poel, Dekker & Langevelde 2015) |  |  |  |
|  |  | Highly ranked | (Pettett *et al.* 2017) |  |  |  |
|  |  | May be attractive | (Hof, Allen & Bright 2019) |  |  |  |
|  |  | Positive link | (Yarnell & Pettett 2020) |  |  |  |
|  |  | Confirmed use | (Berger *et al.* 2020b) |  |  |  |
|  |  | Positive correlation | (Hof 2009) |  |  |  |
|  |  | Strong preference of use | (Dowie 1993) |  |  |  |
| J.2.1.2 | Intact species poor hedge | Positive link | (Micol, Doncaster & Mackinlay 1994) | Negligible | 1 |  |
|  |  | Positive link to edge habitats | (Huijser & Bergers 2000) |  |  |  |
|  |  | Positive association | (Doncaster, Rondinini & Johnson 2001) |  |  |  |
|  |  | Favoured (edge habitats) | (Riber 2006) |  |  |  |
|  |  | Highly selected | (Hof & Bright 2010) |  |  |  |
|  |  | Positive association | (Hof & Bright 2012) |  |  |  |
|  |  | Positive effect | (van de Poel, Dekker & Langevelde 2015) |  |  |  |
|  |  | Highly ranked | (Pettett *et al.* 2017) |  |  |  |
|  |  | May be attractive | (Hof, Allen & Bright 2019) |  |  |  |
|  |  | Positive correlation | (Hof 2009) |  |  |  |
|  |  | Strong preference of use | (Dowie 1993) |  |  |  |
| J.2.2.1 | Defunct species rich hedge | No references | | Negligible | 1 | Proxy intact species poor hedge |
| J.2.2.2 | Defunct species poor hedge | No references | | Negligible | 1 | Proxy intact species poor hedge |
| J.2.3.1 | Species rich hedge with trees | Positive link | (Micol, Doncaster & Mackinlay 1994) | Negligible | 1 |  |
|  |  | Positive link to edge habitats | (Huijser & Bergers 2000) |  |  |  |
|  |  | Positive association | (Doncaster, Rondinini & Johnson 2001) |  |  |  |
|  |  | Favoured (edge habitats) | (Riber 2006) |  |  |  |
|  |  | Highly selected | (Hof & Bright 2010) |  |  |  |
|  |  | Positive association | (Hof & Bright 2012) |  |  |  |
|  |  | Determinant of permeability | (Moorhouse *et al.* 2014) |  |  |  |
|  |  | Positive effect | (van de Poel, Dekker & Langevelde 2015) |  |  |  |
|  |  | Highly ranked | (Pettett *et al.* 2017) |  |  |  |
|  |  | May be attractive | (Hof, Allen & Bright 2019) |  |  |  |
|  |  | Positive link | (Yarnell & Pettett 2020) |  |  |  |
|  |  | Confirmed use | (Berger *et al.* 2020b) |  |  |  |
|  |  | Positive correlation | (Hof 2009) |  |  |  |
|  |  | Strong preference of use | (Dowie 1993) |  |  |  |
| J.2.3.2 | Species poor hedge with trees | Positive link | (Micol, Doncaster & Mackinlay 1994) | Negligible | 1 |  |
|  |  | Positive link to edge habitats | (Huijser & Bergers 2000) |  |  |  |
|  |  | Positive association | (Doncaster, Rondinini & Johnson 2001) |  |  |  |
|  |  | Favoured (edge habitats) | (Riber 2006) |  |  |  |
|  |  | Highly selected | (Hof & Bright 2010) |  |  |  |
|  |  | Positive association | (Hof & Bright 2012) |  |  |  |
|  |  | Determinant of permeability | (Moorhouse *et al.* 2014) |  |  |  |
|  |  | Positive effect | (van de Poel, Dekker & Langevelde 2015) |  |  |  |
|  |  | Highly ranked | (Pettett *et al.* 2017) |  |  |  |
|  |  | May be attractive | (Hof, Allen & Bright 2019) |  |  |  |
|  |  | Positive link | (Yarnell & Pettett 2020) |  |  |  |
|  |  | Confirmed use | (Berger *et al.* 2020b) |  |  |  |
|  |  | Positive correlation | (Hof 2009) |  |  |  |
|  |  | Strong preference of use | (Dowie 1993) |  |  |  |
| J.2.4 | Fence | No references | | Medium | 41 to 60 | Depends on condition and presence of gaps  No proxy |
| J.2.5 | Wall | No references | | High | 81 to 98 | Lower walls could be climbed so pose less of a barrier  No proxy |
| J.2.6 | Dry ditch | No references | | Negligible | 1 | Proxy grassland habitats |
| J.2.7 | Boundary removed | No references | | Negligible | 1 | Proxy grassland habitats |
| J.2.8 | Earth bank | No references | | Negligible | 1 | Proxy grassland habitats |
| J.3.4 | Caravan site | No references | | Medium-high | 61 to 80 | Assuming small garden / amenity areas are present amongst caravans, which is common, although often heavily managed and lit  Proxy buildings and track / road |
| J.3.5 | Sea wall | No references | | Complete | 99 | Proxy buildings |
| J.3.6 | Buildings | Highly ranked | (Pettett *et al.* 2017) | Complete | 99 | Mixed correlations, likely driven by other factors such as gardens, amenity areas, parks etc. |
|  |  | Positive link - fewer badgers, more food | (Williams *et al.* 2018) |  |  |  |
|  |  | Negative relationship | (Hof, Allen & Bright 2019) |  |  |  |
|  |  | Favoured | (Driezen *et al.* 2007) |  |  |  |
|  |  | Favourable habitat | (Wright *et al.* 2020) |  |  |  |
|  |  | Avoided | (Dowie 1993) |  |  |  |
| J.3.7 | Track / road | Negative link | (Micol, Doncaster & Mackinlay 1994) | Medium | 41 to 60 | Likely to vary depending on the size of road and traffic levels, adjacent habitats etc. |
|  |  | Verges used (split into 2) | (Doncaster, Rondinini & Johnson 2001) |  |  |  |
|  |  | Less preferred inc verges, reluctance to cross larger roads | (Rondinini & Doncaster 2002) |  |  |  |
|  |  | 35% lower densities, larger roads bigger barrier effect | (Orłowski & Nowak 2004) |  |  |  |
|  |  | Verges used, large roads bigger barrier | (Hof & Bright 2009) |  |  |  |
|  |  | Clear aversion but would cross during the night | (Dowding *et al.* 2010) |  |  |  |
|  |  | Positive for minor roads, negative for major roads | (Hof & Bright 2012) |  |  |  |
|  |  | Positive effect - likely linked to lower badger numbers | (van de Poel, Dekker & Langevelde 2015) |  |  |  |
|  |  | Positive link - fewer badgers, more food | (Williams *et al.* 2018) |  |  |  |
|  |  | Positive link (verges) | (Yarnell & Pettett 2020) |  |  |  |
|  |  | Smaller roads no resistence, main streets acted as major barriers | (Braaker *et al.* 2014) |  |  |  |
|  |  | Favourable habitat (verges) | (Wright *et al.* 2020) |  |  |  |
|  |  | General avoidance | (Reeve 1981) |  |  |  |
|  |  | Minor road positively correlated (verge habs), major roads negatively correlated | (Hof 2009) |  |  |  |
|  |  | Avoided | (Dowie 1993) |  |  |  |
|  |  | Used in nightly ranging, avoided within home ranges, didn't actively avoid crossing | (Dowding 2007) |  |  |  |
| J.3.7 | Road - including all unpaved and asphalt roads, and railroads. | Negative link | (Micol, Doncaster & Mackinlay 1994) | Low | 2 to 20 | Likely to vary depending on traffic levels, adjacent habitats etc. |
|  |  | Verges used (split into 2) | (Doncaster, Rondinini & Johnson 2001) |  |  |  |
|  |  | Least preferred | (Rondinini & Doncaster 2002) |  |  |  |
|  |  | Lower densities | (Orłowski & Nowak 2004) |  |  |  |
|  |  | Verges used | (Hof & Bright 2009) |  |  |  |
|  |  | Clear aversion but would cross during the night | (Dowding *et al.* 2010) |  |  |  |
|  |  | Positive link | (Hof & Bright 2012) |  |  |  |
|  |  | Positive effect - likely linked to lower badger numbers | (van de Poel, Dekker & Langevelde 2015) |  |  |  |
|  |  | Positive link - fewer badgers, more food | (Williams *et al.* 2018) |  |  |  |
|  |  | Positive link (verges) | (Yarnell & Pettett 2020) |  |  |  |
|  |  | No resistence | (Braaker *et al.* 2014) |  |  |  |
|  |  | Favourable habitat (verges) | (Wright *et al.* 2020) |  |  |  |
|  |  | General avoidance | (Reeve 1981) |  |  |  |
|  |  | Positively correlated | (Hof 2009) |  |  |  |
|  |  | Avoided | (Dowie 1993) |  |  |  |
|  |  | Used in nightly ranging, avoided within home ranges, didn't actively avoid crossing | (Dowding 2007) |  |  |  |
| J.3.7 | Large Road - as above but over 4m wide | Negative link | (Micol, Doncaster & Mackinlay 1994) | High | 81 to 98 | Likely to vary depending on traffic levels, adjacent habitats etc. |
|  |  | Verges used (split into 2) | (Doncaster, Rondinini & Johnson 2001) |  |  |  |
|  |  | Reluctance to cross | Rondinini & Doncaster 2002 |  |  |  |
|  |  | Large barrier effect | (Orłowski & Nowak 2004) |  |  |  |
|  |  | Large barrier effect | (Hof & Bright 2009) |  |  |  |
|  |  | Clear aversion but would cross during the night | (Dowding *et al.* 2010) |  |  |  |
|  |  | Negative link | (Hof & Bright 2012) |  |  |  |
|  |  | Positive effect - likely linked to lower badger numbers | (van de Poel, Dekker & Langevelde 2015) |  |  |  |
|  |  | Positive link - fewer badgers, more food | (Williams *et al.* 2018) |  |  |  |
|  |  | Positive link (verges) | (Yarnell & Pettett 2020) |  |  |  |
|  |  | Barrier effect | (Braaker *et al.* 2014) |  |  |  |
|  |  | Favourable habitat (verges) | (Wright *et al.* 2020) |  |  |  |
|  |  | General avoidance | (Reeve 1981) |  |  |  |
|  |  | Negatively correlated | (Hof 2009) |  |  |  |
|  |  | Avoided | (Dowie 1993) |  |  |  |
|  |  | Used in nightly ranging, avoided within home ranges, didn't actively avoid crossing | (Dowding 2007) |  |  |  |
| J.4 | Bare ground | No references | | Low-medium | 21 to 40 | Will be variable depending on what the habitat is and the extent  Proxy other habitat |
| J.5 | Other habitat | Waste ground favoured | (Hof 2009) | Low-medium | 21 to 40 | Will be variable depending on what the habitat is |
|  |  | Least selected | (Dowding 2007) |  |  |  |
|  | Other notes | Positive link with uplands, explained by roads and badgers | (Hof & Bright 2012) |  |  |  |
|  |  | Absent above tree line | (Williams *et al.* 2018) |  |  |  |
|  |  | Avoided high light intensity | (Berger *et al.* 2020a) |  |  |  |
|  |  | Food availability and connectivity key drivers to presence, larger field sizes likely to hinder movement | (Yarnell & Pettett 2020) |  |  |  |
|  |  | Adjusted behaviour in urban areas, able to respond to temporary disturbance | (Berger *et al.* 2020b) |  |  |  |
|  |  | Connectivity is less important than quality, although connectivity issues create pinch points | (Braaker *et al.* 2014) |  |  |  |
|  |  | Preference for cover over open habs in first year, reverse in second year (due to lack of radio tracking, biased by ability to see animals), clear use of edge habitats even when in open areas | (Reeve 1981) |  |  |  |
|  |  | Selected for upland habs (downs, moors, heaths), agri-environment schemes small positive effect | (Hof 2009) |  |  |  |
|  |  | Habitat use dictated by need to foraging - need to fill stomachs 2/3 times per night | (Dowding 2007) |  |  |  |

**Table S4: Summary of road resistance values by type**

| **Road Type** | **Typical Resistance Level** | **Typical Resistance Value (0 to 99)** | **GIS input value** |
| --- | --- | --- | --- |
| A Road | High | 81 to 98 | 91 |
| B Road | Medium-high | 61 to 80 | 71 |
| Classified Unnumbered | Medium | 41 to 60 | 51 |
| Motorway | Complete | 99 | 99 |
| Not Classified | Low-medium | 21 to 40 | 31 |
| Unclassified | Low-medium | 21 to 40 | 31 |

**Table S5: Summary of watercourse resistance values by type**

| **Watercourse Type** | **Typical Resistance Level** | **Typical Resistance Value (0 to 99)** | **GIS input value** |
| --- | --- | --- | --- |
| Canal | High | 81 to 98 | 91 |
| Inland River | High | 81 to 98 | 91 |
| Lake | Complete | 99 | 99 |
| Tidal River | Complete | 99 | 99 |

**Table S6: Re-genotyping error rate**

| **Locus** | **Total No of Alleles Re-genotyped** | **No of Mismatches** | **Error Rate per Allele** |
| --- | --- | --- | --- |
| E13 | 94 | 0 | 0 |
| EEU1 | 92 | 1 | 0.011 |
| EEU12H | 92 | 0 | 0 |
| EEU37H | 90 | 3 | 0.033 |
| EEU4 | 94 | 0 | 0 |
| EEU2 | 94 | 0 | 0 |
| EEU3 | 96 | 0 | 0 |
| EEU5 | 94 | 0 | 0 |
| EEU6 | 96 | 0 | 0 |
| E36 | 90 | 0 | 0 |
| EEU43H | 96 | 0 | 0 |
| W23 | 88 | 0 | 0 |
| W30 | 98 | 0 | 0 |
| W8 | 98 | 0 | 0 |

**Table S7: Evanno output from Structure Selector**

| # K | Reps | Mean LnP(K) | Stdev LnP(K) | Ln'(K) | \|Ln''(K)\| | Delta K |
| --- | --- | --- | --- | --- | --- | --- |
| 1 | 5 | -11180.26000 | 0.21909 | NA | NA | NA |
| 2 | 5 | -11106.68000 | 18.48004 | 73.58000 | 32.88000 | 1.77922 |
| 3 | 5 | -11065.98000 | 23.47247 | 40.70000 | 46.57556 | 1.98426 |
| 4 | 5 | -11071.85556 | 30.46560 | -5.87556 | 227.42889 | 7.46510 |
| 5 | 5 | -11305.16000 | 120.69351 | -33.30444 | 200.74444 | 1.66326 |
| 6 | 5 | -11337.72000 | 200.27922 | -32.56000 | 18.30000 | 0.09137 |
| 7 | 5 | -11388.58000 | 67.89762 | -50.86000 | 145.38000 | 2.14116 |
| 8 | 5 | -11584.82000 | 116.87616 | -96.24000 | 112.28000 | 0.96067 |
| 9 | 5 | -11893.34000 | 251.70065 | -08.52000 | 131.70000 | 0.52324 |
| 10 | 5 | -12070.16000 | 203.01845 | -76.82000 | NA | NA |

**
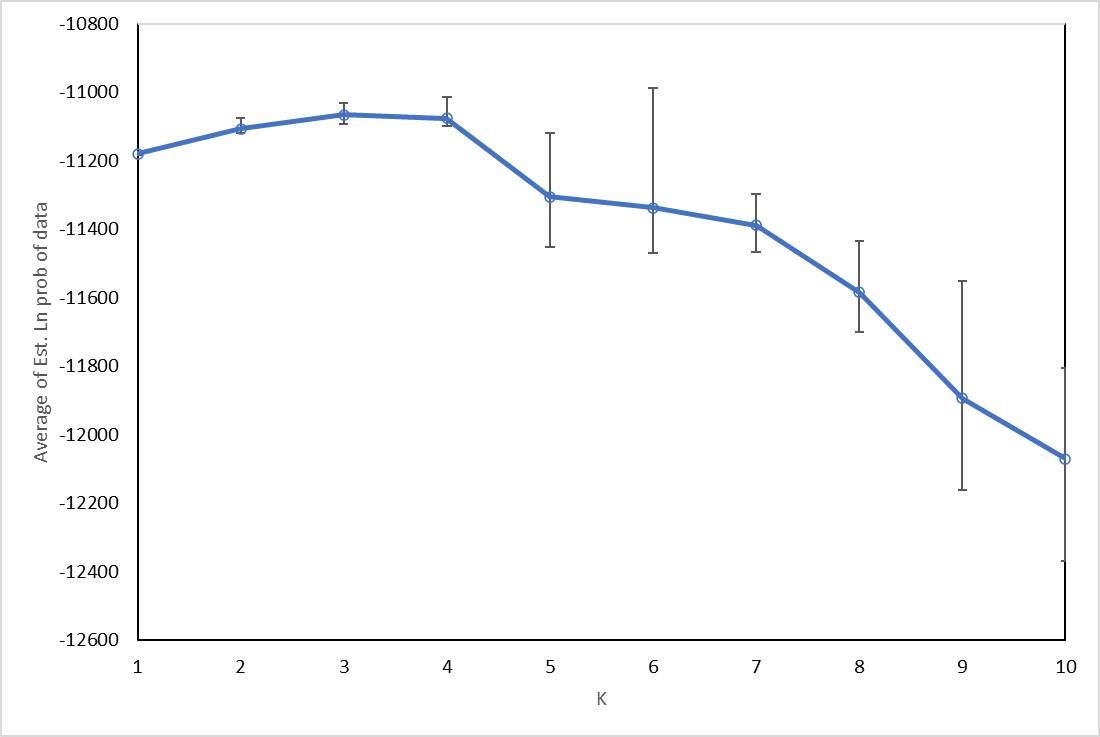
**

**Figure S1: Average Ln Pr(X|K) for K 1-10 across all runs**


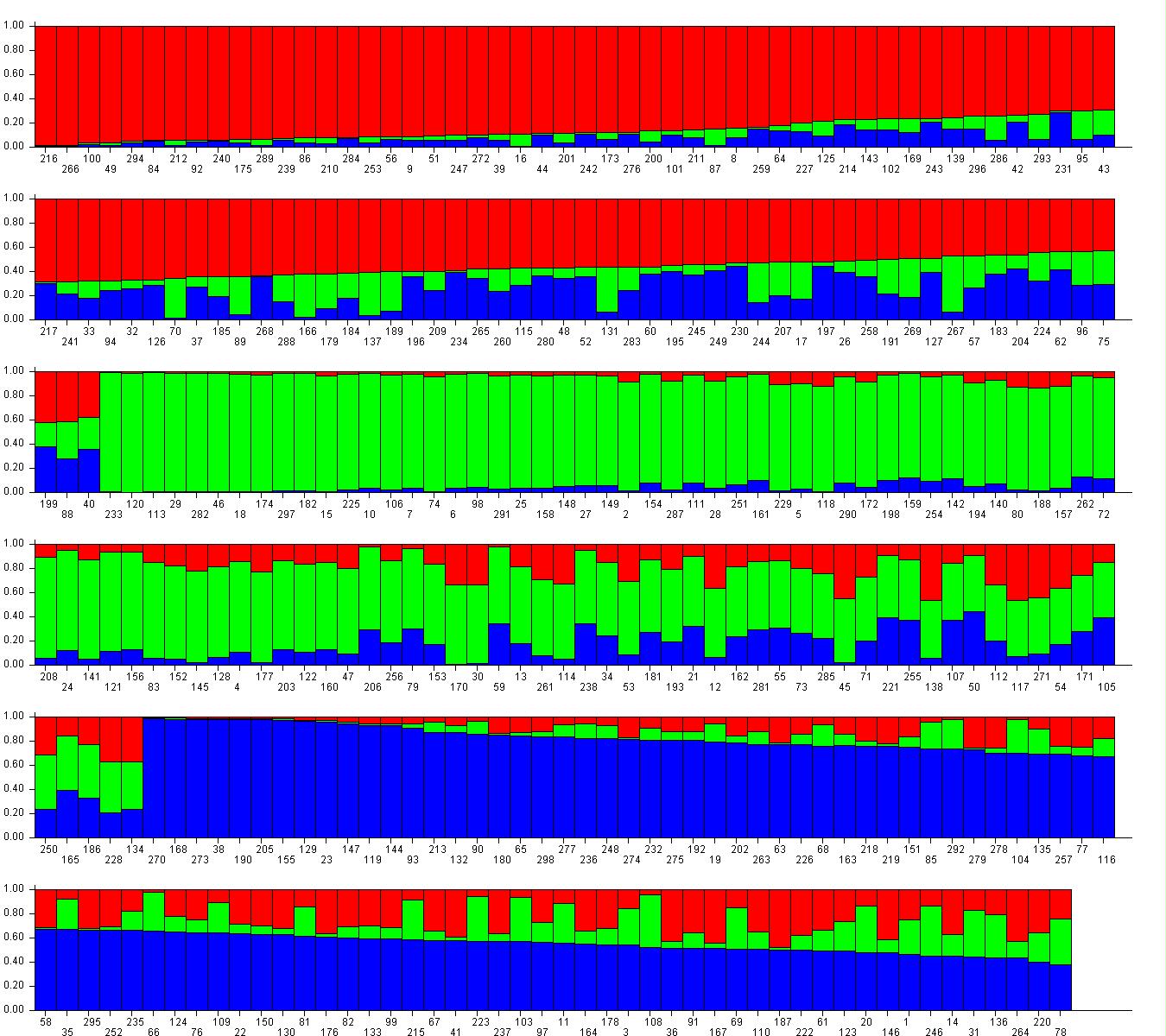


**Figure S2: Results of the genetic structure analysis, proportional ancestry for all samples for three genetic clusters (K=3) as estimated by Structure.**

**Table S8:** **Percentage of genetic variance explained by the retained PCos of different improved grassland habitat resistance values**

| **Variable** | **Variance Explained by Retained PCo (first 10 or 131)** |
| --- | --- |
| Distance | 50.62% |
| Habitats (IG Resistance = 1) | 50.19% |
| Habitats (IG Resistance = 11) | 50.24% |
| Habitats (IG Resistance = 31) | 50.27% |
| Habitats (IG Resistance = 51) | 50.29% |
| Habitats (IG Resistance = 71) | 50.30% |
| Habitats (IG Resistance = 91) | 50.31% |
| Habitats (IG Resistance = 99) | 50.31% |
| Roads | 50.40% |
| Water | 50.63% |
| ***NB:*** *IG resistance = improved grassland resistance which varies within each variable as identified* | |

**Table S9: Results from the dbRDA investigating the impact of resistance variables on pairwise genetic relatedness (Queller and Goodnight (1989) estimator). Displayed are the variables tested, their total variance (inertia), the % variance explained (R^2^) and adjusted % variance explained (adjusted R^2^), the degrees of freedom (df), F-statistic (F), and p-value (Pr(>F)) of the permutation tests (9,999). Models controlled for distance are indicated with |.**

| **Variable** | **Inertia** | **R^2^** | **Adjusted R^2^** | **Df** | **F** | **Pr(>F)** |
| --- | --- | --- | --- | --- | --- | --- |
| Distance | 8.82 | 3.75% | <1% | 10 | 0.995 | 0.688 |
| Roads | 8.83 | 3.76% | <1% | 10 | 0.996 | 0.653 |
| Water | 8.82 | 3.75% | <1% | 10 | 0.995 | 0.693 |
| Habitats (IG Resistance = 1) | 116.20 | 49.43% | <1% | 131 | 1.000 | 0.521 |
| Habitats (IG Resistance = 11) | 116.10 | 49.39% | <1% | 131 | 0.998 | 0.842 |
| Habitats (IG Resistance = 31) | 116.05 | 49.37% | <1% | 131 | 0.997 | 0.886 |
| Habitats (IG Resistance = 51) | 116.02 | 49.36% | <1% | 131 | 0.997 | 0.917 |
| Habitats (IG Resistance = 71) | 116.00 | 49.35% | <1% | 131 | 0.997 | 0.937 |
| Habitats (IG Resistance = 91) | 115.97 | 49.33% | <1% | 131 | 0.996 | 0.957 |
| Habitats (IG Resistance = 99) | 115.98 | 49.34% | <1% | 131 | 0.996 | 0.947 |
|  | | | | | | |
| Roads \| Distance | 8.82 | 3.78% | <1% | 10 | 1.002 | 0.452 |
| Water \| Distance | 8.82 | 3.77% | <1% | 10 | 1.000 | 0.525 |
| Habitats (IG Resistance = 1) \| Distance | 8.82 | 49.40% | <1% | 131 | 0.998 | 0.695 |
| Habitats (IG Resistance = 11) \| Distance | 8.82 | 49.38% | <1% | 131 | 0.997 | 0.751 |
| Habitats (IG Resistance = 31) \| Distance | 8.82 | 49.36% | <1% | 131 | 0.997 | 0.800 |
| Habitats (IG Resistance = 51) \| Distance | 8.82 | 49.36% | <1% | 131 | 0.997 | 0.799 |
| Habitats (IG Resistance = 71) \| Distance | 8.82 | 49.36% | <1% | 131 | 0.997 | 0.793 |
| Habitats (IG Resistance = 91) \| Distance | 8.82 | 49.36% | <1% | 131 | 0.996 | 0.815 |
| Habitats (IG Resistance = 99) \| Distance | 8.82 | 49.37% | <1% | 131 | 0.997 | 0.797 |
|  | | | | | | |
| All variables (IG resistance = 31) | 235.06 | 60.71% | <1% | 161 | 0.998 | 0.723 |

**Table S10: Results from the dbRDA (Lynch and Ritland (1999) estimator). Displayed are the tested models for the Lynch and Ritland 1999 estimator, their total variance (inertia), the % variance explained (R^2^) and adjusted % variance explained (adjusted R^2^), the degrees of freedom (df), F-statistic (F), and p-value (Pr(>F)) of the permutation tests (9,999). Models controlled for distance are indicated with |.**

| **Variable** | **Inertia** | **R^2^** | **Adjusted R^2^** | **Df** | **F** | **Pr(>F)** |
| --- | --- | --- | --- | --- | --- | --- |
| Distance | 1.65 | 3.70% | <1% | 10 | 0.980 | 0.971 |
| Habitats (IG Resistance = 1) | 21.96 | 49.40% | <1% | 131 | 0.999 | 0.843 |
| Habitats (IG Resistance = 11) | 21.93 | 49.35% | <1% | 131 | 0.997 | 0.966 |
| Habitats (IG Resistance = 31) | 21.91 | 49.30% | <1% | 131 | 0.995 | 0.986 |
| Habitats (IG Resistance = 51) | 21.92 | 49.33% | <1% | 131 | 0.996 | 0.966 |
| Habitats (IG Resistance = 71) | 21.92 | 49.33% | <1% | 131 | 0.996 | 0.963 |
| Habitats (IG Resistance = 91) | 21.93 | 49.33% | <1% | 131 | 0.996 | 0.957 |
| Habitats (IG Resistance = 99) | 21.93 | 49.34% | <1% | 131 | 0.996 | 0.948 |
| Roads | 1.65 | 3.70% | <1% | 10 | 0.980 | 0.967 |
| Water | 1.64 | 3.70% | <1% | 10 | 0.980 | 0.971 |
|  | | | | | | |
| Roads \| Distance | 1.64 | 3.76% | <1% | 10 | 0.995 | 0.679 |
| Water \| Distance | 1.64 | 3.76% | <1% | 10 | 0.995 | 0.680 |
| Habitats (IG Resistance = 1) \| Distance | 1.64 | 49.34% | <1% | 131 | 0.994 | 0.934 |
| Habitats (IG Resistance = 11) \| Distance | 1.64 | 49.29% | <1% | 131 | 0.992 | 0.980 |
| Habitats (IG Resistance = 31) \| Distance | 1.64 | 49.25% | <1% | 131 | 0.991 | 0.990 |
| Habitats (IG Resistance = 51) \| Distance | 1.64 | 49.28% | <1% | 131 | 0.992 | 0.977 |
| Habitats (IG Resistance = 71) \| Distance | 1.64 | 49.27% | <1% | 131 | 0.992 | 0.982 |
| Habitats (IG Resistance = 91) \| Distance | 1.64 | 49.28% | <1% | 131 | 0.992 | 0.977 |
| Habitats (IG Resistance = 99) \| Distance | 1.64 | 49.29% | <1% | 131 | 0.992 | 0.969 |
|  | | | | | | |
| All variables (IG resistance = 31) | 44.44 | 60.52% | <1% | 161 | 0.990 | 0.998 |

**References**

Becher, S. & Griffiths, R. (1997) Isolation and characterization of six polymorphic microsatellite loci in the European hedgehog Erinaceus europaeus. *Molecular Ecology,* **6,** 89-90.

Berger, A., Barthel, L.M.F., Rast, W., Hofer, H. & Gras, P. (2020a) Urban Hedgehog Behavioural Responses to Temporary Habitat Disturbance versus Permanent Fragmentation. *Animals,* **10,** 2109.

Berger, A., Lozano, B., Barthel, L.M.F. & Schubert, N. (2020b) Moving in the Dark—Evidence for an Influence of Artificial Light at Night on the Movement Behaviour of European Hedgehogs (Erinaceus europaeus). *Animals,* **10,** 1306.

Braaker, S., Moretti, M., Boesch, R., Ghazoul, J., Obrist, M.K. & Bontadina, F. (2014) Assessing habitat connectivity for ground-dwelling animals in an urban environment. *Ecological Applications,* **24,** 1583-1595.

Curto, M., Winter, S., Seiter, A., Schmid, L., Scheicher, K., Barthel, L.M.F., Plass, J. & Meimberg, H. (2019) Application of a SSR-GBS marker system on investigation of European Hedgehog species and their hybrid zone dynamics. *Ecology and Evolution,* **9,** 2814-2832.

Doncaster, C.P., Rondinini, C. & Johnson, P.C.D. (2001) Field test for environmental correlates of dispersal in hedgehogs Erinaceus europaeus. *Journal of Animal Ecology,* **70,** 33-46.

Dowding, C.V. (2007) An investigation of factors relating to the perceived decline of European hedgehogs (Erinaceus europaeus) in Britain. PhD, University of Bristol.

Dowding, C.V., Harris, S., Poulton, S. & Baker, P.J. (2010) Nocturnal ranging behaviour of urban hedgehogs, Erinaceus europaeus, in relation to risk and reward. *Animal Behaviour,* **80,** 13-21.

Dowie, M.T. (1993) The spatial organisation and habitat use of the European hedgehog Erinaceus europaeus on farmland. PhD, Royal Holloway, University of London

Driezen, K., Adriaensen, F., Rondinini, C., Doncaster, C.P. & Matthysen, E. (2007) Evaluating least-cost model predictions with empirical dispersal data: A case-study using radiotracking data of hedgehogs (Erinaceus europaeus). *Ecological Modelling,* **209,** 314-322.

Henderson, M., Becher, S.A., Doncaster, C.P. & Maclean, N. (2000) Five new polymorphic microsatellite loci in the European hedgehog Erinaceus europaeus. *Molecular Ecology,* **9,** 1949-1951.

Hof, A. & Bright, P. (2010) The value of agri-environment schemes for macro-invertebrate feeders: Hedgehogs on arable farms in Britain. *Animal Conservation - ANIM CONSERV,* **13**.

Hof, A.R. (2009) A Study of the Current Status of the Hedgehog (*Erinaceus europaeus*) and its Declines in Great Britain Since 1960. Ph.D, Royal Holloway, University of London, Egham, UK.

Hof, A.R., Allen, A.M. & Bright, P.W. (2019) Investigating the Role of the Eurasian Badger (Meles meles) in the Nationwide Distribution of the Western European Hedgehog (Erinaceus europaeus) in England. *Animals,* **9,** 759.

Hof, A.R. & Bright, P.W. (2009) The value of green-spaces in built-up areas for western hedgehogs. *Lutra,* **52,** 69-82.

Hof, A.R. & Bright, P.W. (2012) Factors affecting hedgehog presence on farmland as assessed by a questionnaire survey. *Acta Theriologica,* **57,** 79-88.

Huijser, M.P. & Bergers, P.J.M. (2000) The effect of roads and traffic on hedgehog (Erinaceus europaeus) populations. *Biological Conservation,* **95,** 111-116.

Jackson, D.B. (2007) Factors affecting the abundance of introduced hedgehogs (Erinaceus europaeus) to the Hebridean island of South Uist in the absence of natural predators and implications for nesting birds. *Journal of Zoology,* **271,** 210-217.

JNCC (2010) *Handbook for Phase 1 habitat survey – a technique for environmental audit*. JNCC, Peterborough,.

Lynch, M. & Ritland, K. (1999) Estimation of Pairwise Relatedness With Molecular Markers. *Genetics,* **152,** 1753-1766.

Micol, T., Doncaster, C.P. & Mackinlay, L.A. (1994) Correlates of Local Variation in the Abundance of Hedgehogs Erinaceus europaeus. *Journal of Animal Ecology,* **63,** 851-860.

Moorhouse, T.P., Palmer, S.C.F., Travis, J.M.J. & Macdonald, D.W. (2014) Hugging the hedges: Might agri-environment manipulations affect landscape permeability for hedgehogs? *Biological Conservation,* **176,** 109-116.

Orłowski, G. & Nowak, L. (2004) Road mortality of hedgehogs Erinaceus spp. in farmland in lower Silesia (South-Western Poland). **52,** 377-382.

Pettett, C., Moorhouse, T., Johnson, P. & Macdonald, D. (2017) Factors affecting hedgehog (Erinaceus europaeus) attraction to rural villages in arable landscapes. *European Journal of Wildlife Research,* **63**.

Queller, D.C. & Goodnight, K.F. (1989) ESTIMATING RELATEDNESS USING GENETIC MARKERS. *Evolution,* **43,** 258-275.

Reeve, N.J. (1981) A field study of the hedgehog (Erinaceus europaeus) with particular reference to movement and behaviour. PhD, University of London.

Riber, A.B. (2006) Habitat use and behaviour of European hedgehogErinaceus europaeus in a Danish rural area. *Acta Theriologica,* **51,** 363-371.

Rondinini, C. & Doncaster, C.P. (2002) Roads as barriers to movement for hedgehogs. *Functional Ecology,* **16,** 504-509.

van de Poel, J., Dekker, J. & Langevelde, F. (2015) Dutch hedgehogs Erinaceus europaeus are nowadays mainly found in urban areas, possibly due to the negative Effects of badgers Meles meles. *Wildlife Biology,* **21,** 51-55.

Williams, B., Baker, P., Thomas, E., Wilson, G., Judge, J. & Yarnell, R. (2018) Reduced occupancy of hedgehogs (Erinaceus europaeus) in rural England and Wales: The influence of habitat and an asymmetric intra-guild predator. *Scientific Reports,* **8**.

Williams, R.L., Stafford, R. & Goodenough, A.E. (2015) Biodiversity in urban gardens: Assessing the accuracy of citizen science data on garden hedgehogs. *Urban Ecosystems,* **18,** 819-833.

Wright, P.G.R., Coomber, F.G., Bellamy, C.C., Perkins, S.E. & Mathews, F. (2020) Predicting hedgehog mortality risks on British roads using habitat suitability modelling. *PeerJ,* **7,** e8154.

Yarnell, R.W. & Pettett, C.E. (2020) Beneficial Land Management for Hedgehogs (Erinaceus europaeus) in the United Kingdom. *Animals,* **10,** 1566.

Young, R., Davison, J., Trewby, I., Wilson, G., Delahay, R. & Doncaster, C. (2006) Abundance of hedgehogs (Erinaceus europaeus) in relation to the density and distribution of badgers (Meles meles). *Journal of Zoology,* **269**.
